# Studies on the Interactions of 3,11-Difluoro-6,8,13-trimethyl-8*H*-quino[4,3,2-*kl*]acridinium and Insulin with the Quadruplex-Forming Oligonucleotide Sequence a2 from the Insulin-Linked Polymorphic Region

**DOI:** 10.3390/molecules26216595

**Published:** 2021-10-30

**Authors:** Peter Jonas Wickhorst, Heiko Ihmels, Thomas Paululat

**Affiliations:** Department of Chemistry-Biology, Center of Micro- and Nanochemistry and (Bio-)Technology (Cµ), University of Siegen, Adolf-Reichwein-Str. 2, 57068 Siegen, Germany; peter.wickhorst@student.uni-siegen.de (P.J.W.); paululat@chemie.uni-siegen.de (T.P.)

**Keywords:** DNA recognition, ILPR, spectroscopic methods

## Abstract

Recently, several quadruplex-DNA-forming sequences have been identified in the insulin-linked polymorphic region (ILPR), which is a guanine-rich oligonucleotide sequence in the promoter region of insulin. The formation of this non-canonical quadruplex DNA (G4-DNA) has been shown to be involved in the biological activity of the ILPR, specifically with regard to its interplay with insulin. In this context, this contribution reports on the investigation of the association of the quadruplex-forming ILPR sequence **a2** with insulin as well as with the well-known G4-DNA ligand 3,11-difluoro-6,8,13-trimethyl-8*H*-quino[4,3,2-*kl*]acridinium (**1**), also named RHPS4, by optical and NMR spectroscopy. CD- and NMR-spectroscopic measurements confirmed the preferential formation of an antiparallel quadruplex structure of **a2** with four stacked guanine quartets. Furthermore, ligand **1** has high affinity toward **a2** and binds by terminal *π* stacking to the G1–G11–G15–G25 quartet. In addition, the spectroscopic studies pointed to an association of insulin to the deoxyribose backbone of the loops of **a2**.

## 1. Introduction

**Scheme 1:**
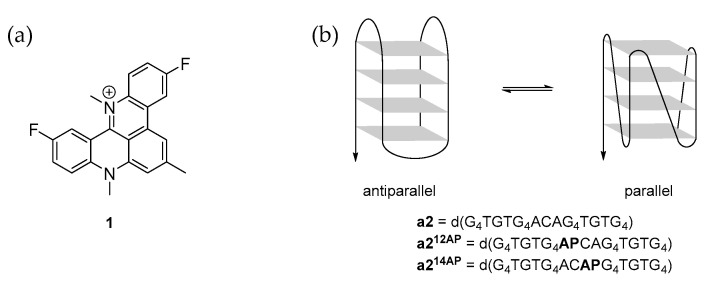
(**a**): Structure of 3,11-difluoro-6,8,13-trimethyl-8*H*-quino[4,3,2-*kl*]acridinium (**1**). (**b**): Equilibrium between antiparallel and parallel conformation of the quadruplex-forming ILPR-DNA sequence **a2** and the 2-aminopurine (**AP**)-labeled sequences **a2^12AP^** und **a2^14AP^**.

Among the various types of biological relevant DNA structures, the non-canonical G-quadruplex DNA (G4-DNA), that is formed in guanine-rich oligo- and polynucleotide sequences, has gained special attention because of its influence on several biological processes such as telomerase inhibition or gene regulation [1,2,3]. For example, in the promotor region, several G-quadruplex-forming gene sequences have been identified that are associated with the transcription of the respective gene, which is likely related to the ability of the corresponding G4-DNA to block unspecific helicases and RNA polymerase or the affinity of G4-DNA toward transcription factors [1,4]. Furthermore, the formation of G4-DNA structures and gene transcription may be regulated by different G4-DNA-binding biomolecules [5,6,7,8,9,10,11]. For example, the formation of the well-studied G4-DNA **c-myc**, which is located in the promoter region of the oncogene *MYC*, is supported by the complexation of nucleolin [5], whereas the interaction with the metastase suppressor NM23-H2 [8] or specific helicases [9,10,11] leads to an unwinding of **c-myc**, which influences the transcription of the gene in both cases. Along these lines, several G4-DNA-forming sequences have been identified within the so-called insulin-linked polymorphic region (ILPR), i.e., a guanine-rich sequence in the promoter region of insulin (Scheme 1), indicating a potential regulation of the insulin expression by G4-DNA [12,13]. Namely, the sequences **a** [d(ACAG_4_TGTG_4_)], **b** [d(ACAG_4_TCTG_4_)], and **c** [d(ACAG_4_TCCTG_4_)] are the most abundant ones in the ILPR. In this series, the sequence **a** exhibits the strongest transcriptional activity [12,13]. Specifically, the sequence **a2** [d(G_4_TGTG_4_ACAG_4_TGTG_4_)] has been the subject of various studies that revealed its ability to adopt a parallel and an antiparallel quadruplex structure, which consist of four guanine quartets, respectively, with different loop structures [14]. Most notably, the results from electrochemical [15], MALDI-MS [16], surface plasmon resonance [16], calorimetric [17], colorimetric [18], fluorimetric [19], and CD-spectroscopic [19] studies indicated that insulin exhibits a high affinity toward **a2**. Therefore, it may be concluded that insulin participates in the formation of quadruplex structures within the ILPR and thus is involved in the regulation of its cellular concentration [1].

G4-DNA structures are also relevant targets for DNA-binding ligands because the formation of quadruplex-ligand complexes may affect the properties of the respective DNA sequence [20]. Hence, it has been shown that small organic ligands influence the formation and stability of G4-DNA structures and thereby govern the physiological function of these DNA forms [1,21,22,23]. In this context, we have investigated the interactions of representative G4-DNA-binding ligands with the ILPR-DNA **a2** and demonstrated that some of them, namely [2,2,2]heptamethinecyanine derivatives and a tetra–azoniapentaphenopentaphene derivative, even exhibit a higher affinity toward this particular G4-DNA structure as compared with other G4-DNA-forming sequences, which points to the potential of small organic ligands to selectively target **a2** [24]. However, to the best of our knowledge, there are no reports on the NMR-spectroscopic investigation of the association of organic ligands with ILPR-DNA, although such studies should reveal more insights in the binding modes. One reason for this knowledge gap may be the limited NMR-spectroscopic data available for ILPR-DNA. As of yet, NMR-spectroscopic studies of the dimeric ILPR sequences and of the particular sequence **a2** have been reported; however, the actual datasets and a complete assignment of the imino proton signals, which is crucial for the assessment of the interactions between ligands and G4-DNA, are not fully available [25].

In our search for suitable ILPR-DNA-targeting ligands, we focused our attention on the well-established acridinium derivative **1** (often referred to as RHPS4). This compound is a very potent G4-DNA-stabilizing agent whose favorable biological properties, such as selective cell growth inhibition, have been assigned to its ability to stabilize telomeric G4-DNA [26]. Furthermore, it was found that ligand **1** binds to promoter G4-DNA structures of the oncogene CD133 and reduces its transcriptional activity in vivo [27]. Moreover, it induces a significant reduction of allergic inflammations in mice, and treatment of tumorous tissues with **1** in combination with ionization radiation even leads to a substantial reduction of the tumor volume, which clearly documents the potential of this substrate as a therapeutic agent in the treatment of G4-DNA-related diseases [28,29]. Therefore, we proposed that this ligand also binds to ILPR-DNA and may even have the ability to form ternary complexes with the ILPR quadruplex and insulin, and thus, it influences the biologically relevant quadruplex–insulin interaction. In this contribution, we present our studies of the interactions of ligand **1** with ILPR-DNA **a2** and with insulin by optical and ^1^H NMR spectroscopy.

## 2. Results

**Figure 1:**
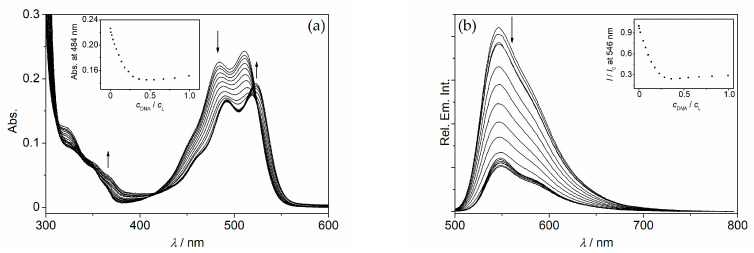
Photometric (**a**) and fluorimetric titration (**b**) of **1** (*c* = 20 μM) with **a2** in K-phosphate buffer (*c*_K_^+^ = 25 mM, pH 7.0); *λ*_ex_ = 485 nm. The arrows indicate the changes of absorption and emission upon addition of DNA. Inset: Plot of the absorption and emission intensity versus DNA concentration.

The interactions of the ligand **1** and insulin with **a2** were studied by photometric and fluorimetric titrations (Figure 1 and Figure S1). During the addition of **a2** to **1**, a decrease and a red shift of the absorption maxima of **1** at 484 nm and 510 nm (∆*λ* = 13 nm) were observed, which resemble the optical responses observed upon the binding of **1** to several G4-DNA-forming mitochondrial DNA strands and clearly indicate the association of the ligand with the oligonucleotide [30]. Furthermore, the strong fluorescence of ligand **1** at 546 nm was quenched upon the addition of **a2**, which is likely caused by a photo-induced electron transfer (PET) from the DNA bases to the bound excited fluorophore [31,32,33,34]. The Scatchard analysis of the binding isotherm obtained from the fluorimetric titration of ligand **1** with **a2** revealed a binding constant for the complex formation (*K*_b_ = 1.4 ± 0.2 × 10^6^ M^–1^) (Figure S2) [35], which is larger than the binding constant of this ligand with telomeric quadruplex DNA (*K*_b_ = 2.25 × 10^5^ M^–1^) [36]. 

To assess whether the presence of insulin influences the photophysical properties of the complex of **a2** and **1**, a spectrometric titration of insulin to a 1:1 mixture of **a2** and **1** was performed (Figure S1). Notably, only very small changes of the absorption and emission spectra were observed, which indicated that ligand **1** was not displaced from its DNA binding site by insulin. Nevertheless, the small, but significant changes, namely an increase of the absorbance and a decrease of the emission intensity of the ligand, may be caused by the formation of a ternary ligand–insulin–**a2** complex. In this case, the marginal changes indicate that the absorption and emission properties of the ligand in the ternary complex do not differ from the ones within the binary ligand–**a2** complex. Such ternary complexes of G4-DNA structures with proteins and ligands have already been reported, but they have not been investigated regarding their photophysical properties so far [37,38].

**Figure 2:**
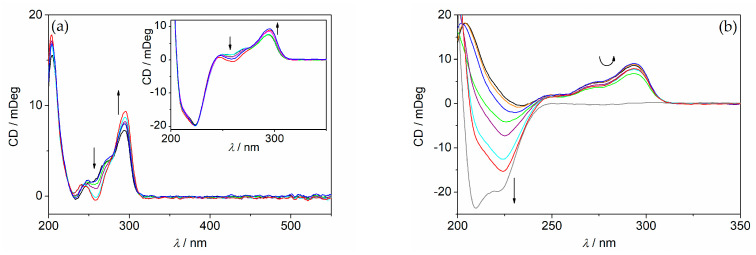
CD spectra of **a2** (*c* = 20 μM) in the absence and presence of **1** (**a**) and insulin (**b**) at a ratio **1**/**a2** = insulin/**a2** = 0 (black), 0.1 (orange), 0.2 (blue), 0.5 (green), 1.0 (purple), 1.5 (cyan), 2.0 (red); in K-phosphate buffer (*c*_K_^+^ = 25 mM, pH 7.0); gray: pure insulin (**b**). The arrows indicate the changes of CD bands with increasing content of **1** (**a**) or insulin (**b**). Inset: CD spectra of a mixture of **a2** and insulin (*c*_DNA_ = 20 μM, *c*_Insulin_ = 20 μM) in the absence and presence of **1** at ligand DNA ratio (*LDR*) = 0 (black), 0.1 (orange), 0.2 (cyan), 0.5 (green), 1.0 (magenta), 1.5 (blue), and 2.0 (red) in K-phosphate buffer (*c*_K_^+^ = 25 mM, pH 7.0).

**Figure 3:**
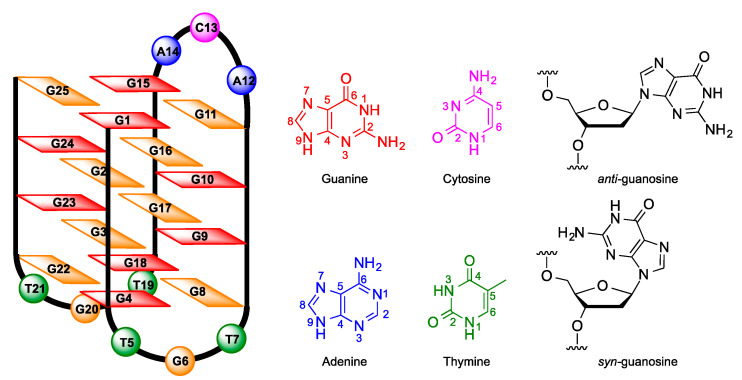
Schematic presentation of the antiparallel G4-DNA structure of **a2** in K-phosphate buffer, the numbering of the four nucleobases guanine, cytosine, adenine, and thymine, and structures of guanosine residues in *syn-* and *anti*-glycosidic conformation; blue: dA, magenta: dC green: dT, red: dG*_syn_*, orange: dG*_anti_*.

Furthermore, the interactions between **1**, insulin, and the ILPR-DNA **a2** were studied by circular dichroism (CD) spectroscopy (Figure 2 and Figure S3). Unfortunately, the oli gonucleotide **a2** is poorly soluble in buffer solution at large ligand and DNA concentrations (c_Ligand_ > 500 µM, c**_a2_** = 2.0 mM), but the solubility was greatly improved at lower ionic strength. To identify the optimal conditions with respect to the solubility and stability of the oligonucleotide, the CD spectra of the DNA were recorded at different K^+^ concentrations. In general, no significant changes of the CD pattern of **a2** were observed in a concentration range between 25 and 100 mM of KCl, indicating no significant structural changes over this range. Therefore, a concentration of 25 mM was selected for further CD-spectroscopic investigations (Figure S3), because no serious solubility issues were observed at this concentration. At these conditions, the oligonucleotide **a2** exhibits a positive CD signal at 290 nm as well as a shoulder at 260 nm. This CD pattern indicates a preferred formation of an antiparallel G4-DNA structure (Figure 3), because according to the literature data, the signal at 290 nm is related to an antiparallel conformation and the signal at 260 nm is related to a parallel one [14,24,25]. Upon addition of the ligand **1** to **a2**, a decrease of the shoulder at 260 nm was observed alongside an increase of the signal at 290 nm, which indicates a shift of the equilibrium from the parallel to the antiparallel structure (Scheme 1) [39]. Interestingly, a similar behavior was observed with telomeric quadruplex DNA in the presence of **1**, which proposedly indicates a general binding preference of the ligand to antiparallel G4-DNA [40]. Moreover, induced CD (ICD) signals in the absorption range of the ligand, which may result from coupling between the transition dipole moments of the DNA bases and the ligand [41,42], were not observed during the titration, which is commonly interpretated as an indication for terminal stacking. However, it should be noted that the absence of a CD signal is certainly no proof of this particular binding mode [43,44].

The addition of insulin to **a2** led to a decrease of the CD signals of **a2** of approximately 20% until a protein–DNA ratio of 0.5 was reached, and subsequently, the signal intensity increased until a ratio of 2.0 was reached. These results confirm those reported previously for a mixture of insulin and **a2** in a ratio of 7:1 [19] and point to the association of insulin with **a2** [15,25,45]. Following that, the interaction of ligand **1** and the insulin–**a2** complex was also followed by CD spectroscopy (Figure 2). The CD pattern of the insulin–**a2** mixture did not differ substantially during the titration of **1** as compared with the one obtained on the titration of **1** to **a2**, which indicates that the complex between **1** and **a2** is not substantially influenced by the presence of insulin.

**Figure 4:**
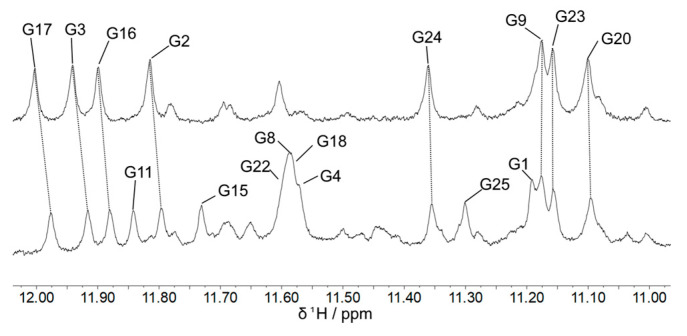
^1^H NMR spectrum of the imino protons (11.0–12.0 ppm) of **a2** (*c* = 2.0 mM) in K-phosphate buffer (*c*_K_^+^ = 25 mM, pH 7.0, T = 25 °C) before (lower panel) and directly after exchange of the solvent from H_2_O/D_2_O (9/1) to D_2_O (upper panel).

To investigate the binding of **1** and insulin to ILPR-DNA **a2** in more detail, NMR-spectroscopic measurements were performed. For this purpose, the NMR signals of the oligonucleotide **a2** had to be assigned based on the ^1^H-, ^1^H-^13^C HSQC, ^1^H-^13^C HMBC, and NOESY NMR spectra in K-phosphate buffer (Table S1). In a first orienting experiment, the ^1^H NMR spectra of **a2** were recorded in H_2_O/D_2_O (9:1) at varying temperature between 21 and 35 °C in order to determine the temperature at which an optimal separation of the guanine H1 proton signals was achieved (Figure S4). The best separation was observed at 25 °C, which was selected for the following NMR-spectroscopic investigations. In the range between 11.10 and 11.98 ppm, 16 guanine imino proton signals were detected (Figures 4 and 6), which revealed the formation of four guanine quartets. In combination with the results of the CD-spectroscopic analysis (see above), it was deduced that an antiparallel structure with four guanine quartets, two TGT loops, and one ACA loop is formed as the major conformation of **a2** (Figure 3). Notably, the relatively small full width at half maximum (FWHM) of ≈6 Hz for guanine H1 proton bands and 2–4 Hz for aromatic proton signals indicated that this set of signals originates from a monomeric quadruplex structure [46,47]. Nevertheless, an additional set of signals was observed with comparatively low signal intensity (rel. Int. *I*_11.64 ppm_/*I*_11.73 ppm_ = 0.3), which may originate from a minor parallel conformation [24]. In order to identify the readily exchangeable guanine H1 protons of the terminal quartets and distinguish them from the ones in the interior of the quadruplex, a H–D exchange experiment [46] was performed (Figure 4). The signals at 11.19, 11.30, 11.57–11.60, 11.73, and 11.84 ppm disappeared or at least their relative intensity decreased immediately after exchange of the solvent from H_2_O/D_2_O (9/1) to D_2_O. Accordingly, these signals were assigned to the protons of the terminal quartets, as they are more easily accessible by the solvent and more prone to H–D exchange. Contrarily, the signals at 11.10, 11.16, 11.18, 11.35, 11.80, 11.88, 11.92, and 11.98 ppm remained essentially unchanged, although slightly weaker, after the addition of D_2_O and were therefore assigned to the guanine H1 protons of the inner quartets, which are shielded more efficiently from the solvent.

**Figure 5:**
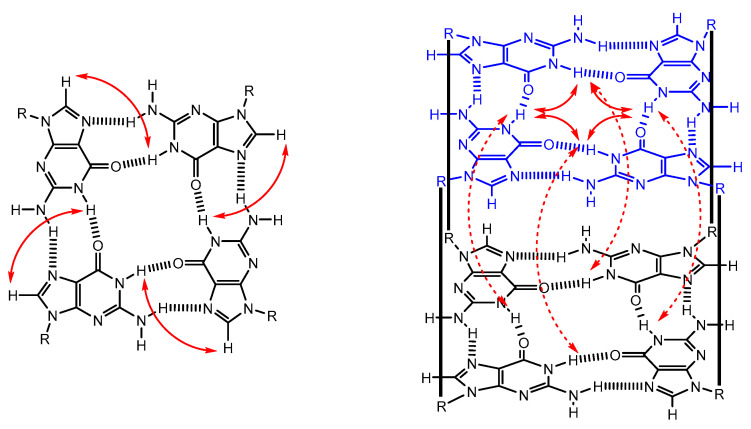
Schematic representation of the observed interbase guanine H1/H8 signals and of the NOE signals between guanine H1 protons (dashed arrows).

**Figure 6:**
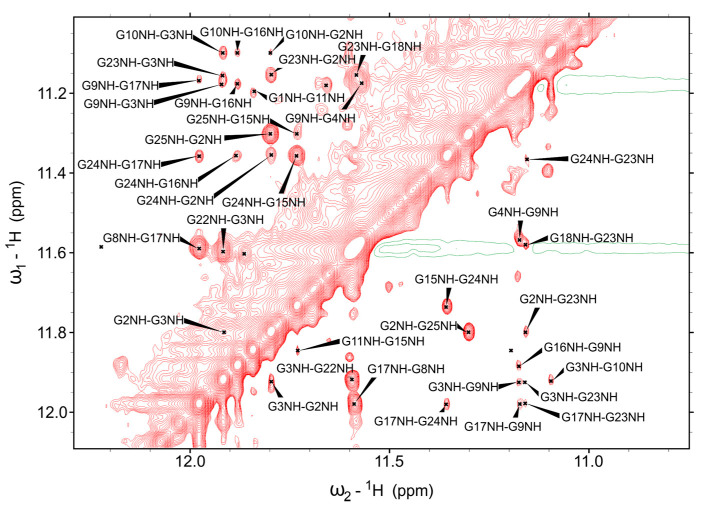
Expansion of the 2D NMR spectrum (NOESY) of the resonances of guanine H1 protons (11.0–12.0 ppm) of **a2** (c = 2.0 mM) in K-phosphate buffer (*c*_K_^+^ = 25 mM, pH 7.0, H_2_O/D_2_O 9:1, *T* = 25 °C).

The signals of the guanine imino protons of G1, G11, G15, and G25 were assigned based on the observed intraquartet NOESY cross-peaks between adjacent bases (Figure 5, Figure 6 and Figure S5). Accordingly, the remaining imino proton signals at 11.57–11.60 ppm were assigned to G4, G8, G18, and G22 of the terminal quartet. Furthermore, strong interquartet NOE signals between the guanine H1 protons of one quartet and the guanine H1 protons of the neighboring quartet as well as the intraquartet NOE signals between two adjacent guanine bases were used to assign the signals of the remaining two tetrads (Figure 5, Figure 6 and Figure S5) [48,49].

The H1/H8 connectivity between the guanine bases and assignment of H8 proton signals (Figure S8) were obtained starting from G8 and the strong NOE cross-peaks between T7Me and G8H1 and G8H1’, with the latter being related to G8H8. Furthermore, the proton G4H1 exhibited a strong NOE signal to G8H8 so that a G4 → G8 → G18 → G22 connectivity was deduced, which led to the remaining guanine H8 proton signals. To add to that, the conformation of the glycosidic bond of each base was obtained from the characteristic ^13^C NMR signals at low field of a syn-guanine-C2 [50] determined from HSQC spectra (Figure S6), which were further supported by the relatively stronger H1’/H8 NOE signals of guanine bases in syn glycosidic conformation as compared with the ones of the anti-orientated guanine bases (Figure S9B) [49]. As a result, the guanine H8 proton signals as well as the conformation of the glycosidic bonds of all guanine bases were determined. Notably, the NOE cross-peaks and glycosidic bond conformations were only in agreement with an interquartet syn-anti-anti-syn conformation [48,49]. Finally, the strong H8/H1’, H8/H2’, and H1’/H2’ NOE signals enabled the assignment of most of the H1’ and H2’/H2’’ protons as well, which in turn gave access to an assignment of the H3’, H4’, and H5’/H5’’ proton signals (Figures S9–S11) [49]. However, it should be noted that due to the strong overlap between the deoxyribose proton signals, the assignment remained incomplete. 

Next, the proton signals of the nucleotides in the loops of **a2** were assigned. The signals of adenine, cytosine, and thymine units were identified from their characteristic shifts and cross-peaks in the HSQC and HMBC spectra (Figures S6 and S7, Table S1). To start with, the adenine C2 exhibits a downfield-shifted signal in the ^13^C NMR spectrum, which enabled the unambiguous assignment of the adenine H2 proton signals with HSQC spectra [48,49]. Likewise, the cytosine H5 and H6 proton signals were assigned from HSQC and HMBC spectra based on the correlation with the characteristic ^13^C NMR chemical shift of the cytosine C5 signal. This assignment was supported by the strong NOESY cross-peaks between C13H5 and C13H6. Accordingly, the proton signals of the thymine methyl groups were assigned from their upfield-shifted ^13^C NMR signals in the HSQC spectra, which were in turn used to assign the respective thymine H6 signal from the strong cross-peaks in the HMBC and NOESY spectra. Finally, the aromatic proton signals of the adenine bases were assigned by means of the H2/H1’ and H8/H1’ NOE connectivity, which was followed by an assignment of most adenosine, cytosine, and thymidine deoxyribose proton signals. The internucleotide NOE cross-peaks between A12/G11, A12/C13, A14/C13, and A14/G15 were detected in combination with the ones between A12H8/G1H1 and A12H2 and all guanine H1 proton signals of the G1–G11–G15–G25 quartet (Figures S8 and S9B), indicating the stacking of A12 on top of the G1 base with the A12H2 proton located in essentially a central position above the quartet. Furthermore, the absence of NOE signals of A14 related to the guanine imino proton signals indicated that A14 might protrude partly into the groove. Unfortunately, the assignment of the proton signals of the TGT loops turned out to be difficult due to a strong overlap of the TMe and TH6 signals and the absence of NOE signals between T5, T19, and T21 and the H1 and H8 proton signals of the guanines. Nevertheless, several NOE cross-peaks between T7 and G8 were detected, which indicated that T7 stacks above G8. Furthermore, the T7Me/T5H2’/H2” NOE interactions as well as NOE signals between G6 and T7 and T5 indicated that T5, G6, and T7 are connected within the same loop, so a T5/G4 connection is proposed. However, no NOE cross-peaks between T19/G18 and T21/G22 were detected, so this loop could not be assigned unambiguously. Nevertheless, NOE interactions between T19 and G8 suggested a close proximity of these nucleotides and a corresponding T19/G18 connectivity. Notably, NOE signals were determined for G20H8/T19H4’ and G20H3’/T21H6 that prove the connectivity of these bases within the same loop. Finally, the absence of NOE cross-peaks between T21 and all guanine bases indicated that this thymine base might protrude out of the guanine quartet.

**Figure 7:**
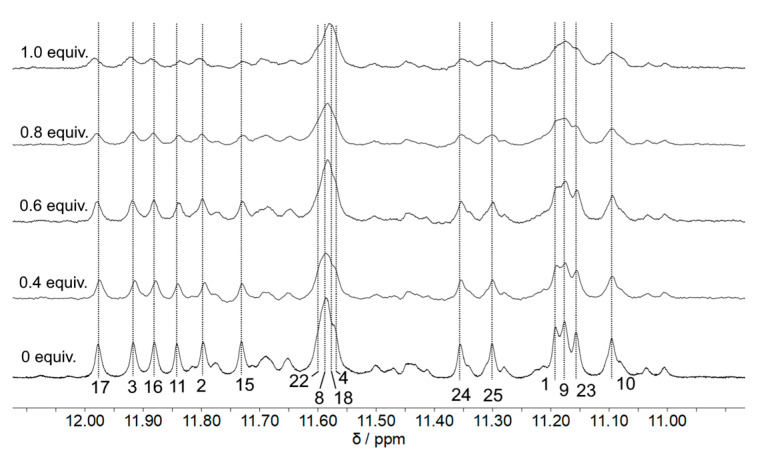
^1^H NMR spectrum of **a2** (*c* = 2.0 mM) in the range of the guanine imino protons (11.0–12.0 ppm) with increasing amount of ligand **1** in K-phosphate buffer (*c*_K_^+^ = 25 mM, pH 7.0, *T* = 25 °C).

**Figure 8:**
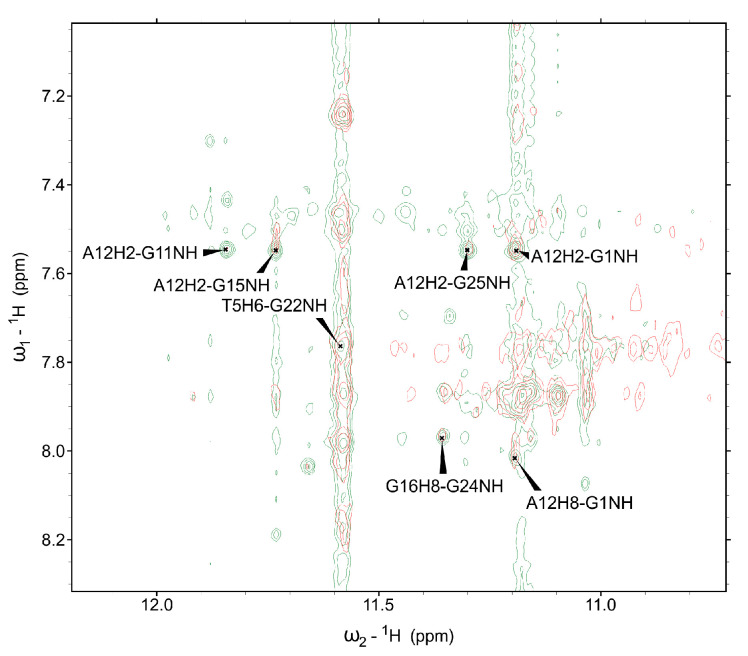
Superposed 2D NMR spectra (NOESY) of guanine imino protons of **a2** (*c*_DNA_ = 2.0 mM) in the absence (green) and presence (red) of 0.6 molar equivalent ligand **1** in K-phosphate buffer [*c*_K_^+^ = 25 mM, pH 7.0, H_2_O/D_2_O (9:1), *T* = 25 °C].

Once the NMR signals of the oligonucleotide **a2** were assigned as far as possible with the available NMR data, the association of ligand **1** with **a2** was studied in more detail by NMR-spectroscopic experiments (Figure 7, Figure 8 and Figure S12). First of all, all proton signals of **a2** broadened upon the addition of ligand **1** (Figures S13 and S14). Most notably, the intensity of the G1H1, G2H1, G11H1, G15H1, and G25H1 proton signals were affected more by the association of ligand **1** than all other H1 proton resonances, and the signal intensities of the G4–G8–G18–G22 quartet were essentially independent of the ligand concentration. Moreover, the changes of the ^1^H NMR signal intensity of the aromatic proton signals revealed a similar pattern as observed for the imino protons (Figure S13). The strongest changes were observed for the aromatic protons of A12H2 and A14H2, whereas the signals of T19H6 and G8H8 did only change to a lesser extent. Nevertheless, the signal intensity of only few aromatic proton resonances could be analyzed due to the strong overlap of most of these signals. Furthermore, small shifts of all guanine imino proton signals were observed, which showed a similar trend with the most pronounced changes for G1H1 (∆*δ*_H_ = 2.7 Hz) and G11H1 (∆*δ*_H_ = 3.4 Hz). Overall, the changes of the proton resonances of **a2** upon the addition of ligand **1** clearly revealed that the G1–G11–G15–G25 quartet is affected to a larger extent by the presence of ligand **1** than all other guanine bases, which suggests that ligand **1** binds preferably by terminal stacking to this quartet. Moreover, the aromatic proton signals of the ACA loop showed the strongest changes of the signal intensity as clear evidence that it participates in the ligand binding. However, the signals of ligand **1** could not be detected in the presence of **a2**, indicating the presence of several binding modes to **a2**, which is in good agreement with the absence of isoelliptic points during polarimetric titrations. Nevertheless, these results may also indicate that the interaction between **1** and **a2** falls into the intermediate exchange regime [49,51]. 

**Figure 9:**
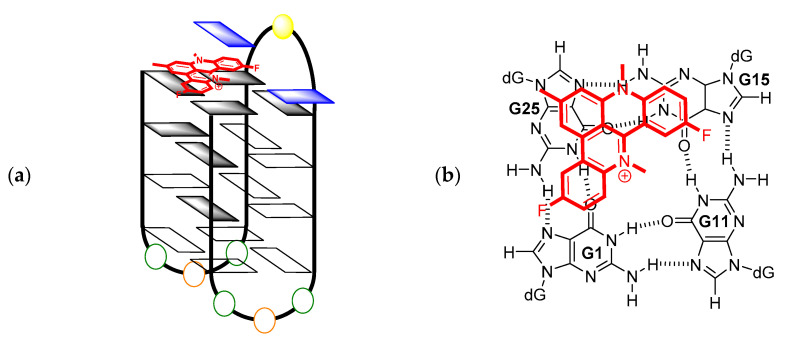
Side view (**a**) and top view (**b**) of the proposed binding mode of ligand **1** at the terminal quartet of **a2**. DNA bases whose NMR signals were influenced strongly by the presence of ligand **1** are shaded.

The binding of ligand **1** to **a2** was also analyzed by NOESY NMR experiments (Figure 8, Figures S15 and S16). As a general trend, no significant shifts of the NOESY cross-peaks of the guanine imino protons were observed (Figure S15). Moreover, the NOE signals between G8H1/T7Me, G8H1’/T19H6, and G8H1’/G6H8 remained essentially unchanged upon the association of **a2** with the ligand (Figure S16), which indicates that these loops are not involved directly in the binding event. In contrast, most of the initial NOE signals between the G1–G11–G15–G25 quartet and the ATA loop decreased strongly (Figure 8), except for the ones of A12H8/G1H1 and A12H2/G1H1, which remained mainly unaffected by the presence of ligand **1**. These observations suggest that A12 is still in close proximity to G1 in the ligand–DNA complex, whereas it becomes more separated from G15 and G25 in the presence of the ligand. Therefore, it is concluded that the ligand approaches the quadruplex at the G25–G15 site of the quartet and thereby partly dislocates A12 from the center of the quartet (Figure 9 and Figure S20). Among the possible geometries to accomplish this binding mode, we assumed a stacking of the ligand atop the triad G1, G15, and G25 as the most likely binding mode because it shows the least steric repulsion between ligand and quadruplex loops. Furthermore, this proposed binding mode by terminal stacking is in good agreement with a similar binding mode of this ligand with G4-DNA **Tel7** [52], even though it is not supported by NOE signals between ligand and DNA.

**Figure 10:**
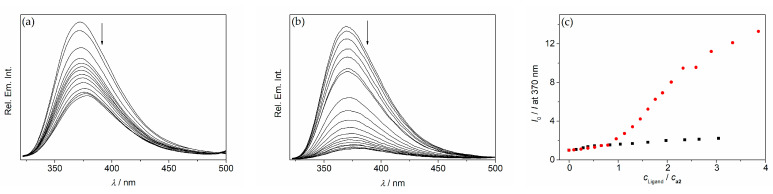
Fluorimetric titration of ligand **1** to **AP**-labeled oligonucleotides **a2^12AP^** (**a**) and **a2^14AP^** (**b**) in K-phosphate buffer (*c*_K_^+^ = 25 mM, pH 7.0); *λ*_ex_ = 305 nm, *c***_a2_** = 30 µM. The arrows indicate the changes of emission upon the addition of **1**. (**c**): Plot of the relative emission *I*_0_/*I* versus *LDR* during the titration with **a2^12AP^** (black squares) and **a2^14AP^** (red circles).

The association of ligand **1** with ILPR DNA **a2** was also followed by fluorimetric titrations with the modified oligonucleotides **a2^12AP^** and **a2^14AP^**, in which an adenine residue in position 12 and 14 was substituted with a 2-aminopurine (**AP**) unit, respectively (Figure 10). The fluorescence of the base **AP** is efficiently quenched when it is π stacked to the neighboring DNA bases in a quadruplex DNA, whereas it has a relatively intense fluorescence in its unstacked and unpaired state. Hence, the relative emission intensity of **AP**-containing oligonucleotides correlates directly with the degree of π stacking and can give valuable information about the molecular environment of **AP** within the DNA structure [53]. This effect is frequently employed for the fluorimetric investigation of the binding of a ligand to an **AP**-labeled DNA strand [54,55]. In aqueous buffer solution, the emission of the oligonucleotide **a2^14AP^** is three times higher than that of **a2^12AP^**, which is likely the result of the close stacking of 12**AP** on top of G1 and G11 [53]. Moreover, the addition of ligand **1** led to fluorescence quenching of both labeled DNA strands, which was caused by the proposed π stacking to the G1–G11–G15–G25 quartet. The observation that the quenching effect is more pronounced for an **AP** in position 14 than in 12 is in agreement with the proposed binding mode (Figure 9). Moreover, the absence of a fluorescence light-up effect shows that the adenine residues are not displaced from the binding site by the ligand and remain available for π stacking. It should be noted that the changes of the fluorescence intensity during the titration were relatively small until a ligand–DNA ratio (*LDR*) of 1, and a significant quenching of the emission of was observed only at higher ratios (Figure 10c). This effect may be caused by the equilibrium between the different quadruplex forms that changes at these ratios, as shown by the CD-spectroscopic analyses (Figure 2).

**Figure 11:**
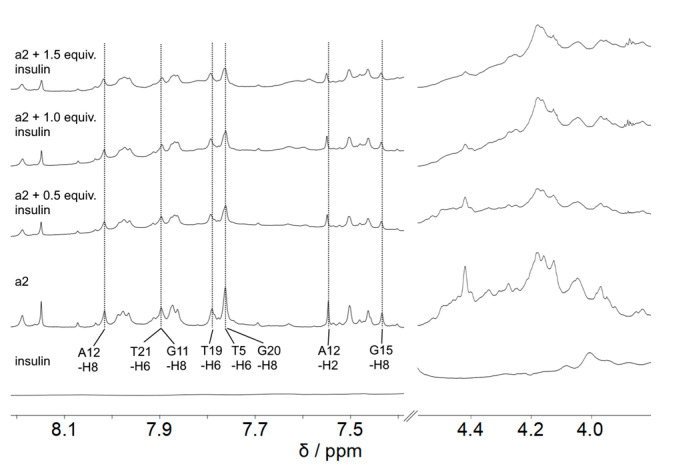
^1^H NMR spectrum of the guanine imino protons (11.0–12.1 ppm) and aliphatic protons (3.75–4.60 ppm) of insulin and **a2** (*c*_DNA_ = 2.0 mM) with increasing amount of insulin in K-phosphate buffer (*c*_K_^+^ = 25 mM, pH 7.0, *T* = 25 °C).

The interaction of insulin with **a2** was also analyzed by ^1^H NMR-spectroscopic studies (Figure 11, Figures S17 and S18). Thus, the signals of the guanine imino protons as well as the aromatic protons of most of the nucleic bases of the guanine tetrads did not change significantly with increasing amounts of insulin (Figures S17 and S18), indicating that the protein does not interact strongly with the quartets of **a2**. Yet, some aromatic proton signals of **a2** changed slightly upon the addition of insulin (Figure 11 and Figure S19). Namely, the chemical shifts of the protons of A12, T19, and T21 were affected slightly by the presence of insulin (∆*δ*_H_ = 1.2–2.2 Hz), whereas nearly all other signals remained essentially unchanged, indicating an interaction of the protein with the ACA and TGT loops of **a2**. However, as this interpretation is based on only small changes of very few signals, it remains rather uncertain. Moreover, the intensity of the deoxyribose proton signals in the region between 4.10 and 4.60 ppm, where proton signals from insulin do not overlap, broadened and decreased substantially (Figure 11). This finding may indicate a weak association of insulin with **a2** and a preferred interaction with the deoxyribose backbone instead of the nucleic bases. However, as the deoxyribose proton signals of **a2** strongly overlap, no specific information about the exact binding mode could be obtained with this set of data. Nevertheless, as it was found that solely the chemical shifts of the aromatic protons of the loop regions changed, it is concluded that the protein binds to the deoxyribose backbone of the ACA and/or TGT loops.

## 3. Materials and Methods

### 3.1. Equipment

Absorption spectra: Varian Cary 100 Bio Spectrophotometer with baseline correction. Emission spectra: Varian Cary Eclipse at 20 °C: Cuvettes: Quartz cells (10 mm × 4 mm). NMR spectra: Varian VNMR-S 600 spectrometer (^1^H: 600 MHz, ^13^C: 150 MHz), equipped with 3 mm and 5 mm triple resonance inverse probes. NMR spectra were processed with the software MestReNova (Santiago de Compostela, Spain; ^1^H NMR, HSQC, HMBC) and Sparky (NOESY) [56]. Circular-dichroism (CD) spectra: Chirascan CD spectrometer, Applied Photophysics. 

### 3.2. Materials

3,11-Difluoro-6,8,13-trimethyl-8*H*-quino[4,3,2-*kl*]acridinium methanosulfate (**1**) was purchased from Selleckchem (Houston, TX, USA) and used without further purification. Insulin was purchased from SigmaAldrich (St. Louis, MO, USA) and used without further purification (Figure S21). Oligodeoxyribonucleotide d[G_4_TGTG_4_ACAG_4_TGTG_4_] (**a2**), d[G_4_TGTG_4_**AP**CAG_4_TGTG_4_] (**a2^12AP^**), and d[G_4_TGTG_4_AC**AP**G_4_TGTG_4_] (**a2^14AP^**) were purchased from Biomers (Ulm, Germany). 

Solutions of oligonucleotide **a2** were prepared in K-phosphate buffer (pH 7), heated to 95 °C for 5 min, and cooled slowly to room temperature within 4 h. K-phosphate buffer: 12.5 mM K_2_HPO_4_; adjusted with 12.5 mM KH_2_PO_4_, 12.5 mM KCl to pH 7.0. All buffer solutions were prepared from purified water (resistivity 18 MΩ cm) and biochemistry-grade chemicals. The buffer solutions were filtered through a PVDF membrane filter (pore size 0.45 μm) prior to use.

^1^H NMR, ^1^H-^13^C HSQC, ^1^H-^13^C HMBC, and NOESY spectra were recorded in 3 mm (150 μL) or 5 mm (500 μL) NMR tubes. One-dimensional (1D) NMR spectra were recorded with solvent suppression with the pulse sequence WET1d with 1.5 s of relaxation time and 256 scans. Solvent suppression in 2D NMR measurements was accomplished with WET solvent suppression, with a mixing time of 300 ms, relaxation time of 1.5 s, 128 scans (HSQC, NOESY) or 512 scans (HMBC), and detection of 2048 × 2048 data points (NOESY) or 2048 × 1024 data points (HSQC, HMBC). Chemical shifts of ^1^H NMR spectra are given in ppm (*δ*) relative to 4,4-dimethyl-4-silapentane-1-sulfonic acid (DSS, *δ* = 0.00 ppm). For ^1^H NMR-spectroscopic monitoring of H–D exchange processes, the oligonucleotide **a2** was repeatedly dissolved in E-pure water and lyophilized (4 times). D_2_O was added directly before the ^1^H NMR measurement.

## 4. Conclusions

In summary, it was confirmed with complementary spectroscopic methods that the quadruplex-forming ILPR-DNA sequence **a2** forms an antiparallel quadruplex structure as the main conformation. Furthermore, a nearly complete assignment of the proton signals was accomplished, which complements the data from earlier studies [25] and provides a useful set of data for future NMR-spectroscopic studies with this G4-DNA. Notably, the CD- and NMR-spectroscopic analysis indicated that insulin binds to this quadruplex DNA. Although the underlying NMR-spectroscopic changes during complex formation are relatively small, they supposedly indicate a weak association of the insulin with the DNA backbone of the deoxyribose backbone of the ACA and/or TGT loops. Therefore, this result provides further insight into the binding mode of insulin with **a2**. Moreover, it was demonstrated that the established G4-DNA ligand **1** binds also to the ILPR-DNA **a2** with high affinity by terminal π stacking to the G1–G11–G15–G25 quartet. The formation of a strong and unambiguously detectable ternary complex between **a2**, insulin, and **1** was not observed. Nevertheless, the overall results may be utilized for the further development of ILPR-DNA-targeting ligands. 

## Data Availability

Data are available from the authors.

## Supplementary Materials

The following items are available online, Figure S1: Photometric and fluorimetric titration of a **1**–**a2** mixture with insulin, Figure S2: Fitting curves of binding isotherms from photometric titrations of **1** with **a2**, Figure S3: CD spectra of **a2** at varying K^+^ concentrations, Figure S4: ^1^H NMR spectra of **a2** at varying temperature, Figure S5: Schematic representation of the observed NOE signals, Figure S6: ^1^H-^13^C HSQC NMR spectra of **a2**, Figure S7: ^1^H-^13^C HSQC and ^1^H-^13^C HMBC NMR spectra of **a2**, Figure S8, Figure S9, Figure S10, Figure S11: Two-dimensional (2D) NOESY NMR spectra of **a2**, Table S1: Signal assignment of **a2**, Figure S12: ^1^H NMR spectra of **a2** at varying concentrations of **1**, Figure S13, Figure S14: Histogram with changes of the signal intensity and chemical shift of the aromatic and imino protons of **a2** in the presence of **1**. Figure S15, Figure S16: 2D NOESY NMR spectra of **a2** in the absence and presence of **1**, Figure S17, Figure S18: ^1^H NMR spectra of **a2** at varying concentrations of insulin, Figure S19: Change of the chemical shift of the aromatic and imino protons of **a2** in the presence of insulin, Figure S20: Binding modes of **1** to **a2**, Figure S21: ^1^H NMR spectrum of ligand **1**. References [35,57] are cited in the supplementary materials.

## Sample Availability

Samples of the compounds are not available from the authors.
