# Supplementary material for: Studies on the Interactions of 3,11-Difluoro-6,8,13-trimethyl-8H-quino[4,3,2-kl]acridinium and Insulin with the Quadruplex-Forming Oligonucleotide Sequence a2 from the Insulin-Linked Polymorphic Region
Source: Molecules. 2021 Oct 30;26(21):6595. doi: 10.3390/molecules26216595 (PMC8587938; doi:10.3390/molecules26216595)
Supplement: Supplementary file 1 [file molecules-26-06595-s001.zip › molecules-1407600-supplementary.pdf]

Studies on the interactions of 3,11-difluoro-6,8,13-trimethyl-8*H*-quino[4,3,2-*kl*]acridinium and insulin with the quadruplex-forming oligonucleotide sequence a2 from the insulin-linked polymorphic region

Peter Jonas Wickhorst [1], Heiko Ihmels [1,*] and Thomas Paululat [1]

Supporting Information

Table of Contents

1. Photometric and fluorimetric DNA titrations S2

2. CD-spectroscopic analysis S3

3. NMR-spectroscopic analysis S4

4. References S20

1. Photometric and fluorimetric DNA titrations

Photometric and fluorimetric titrations with **a2** were performed according to published procedures [1].

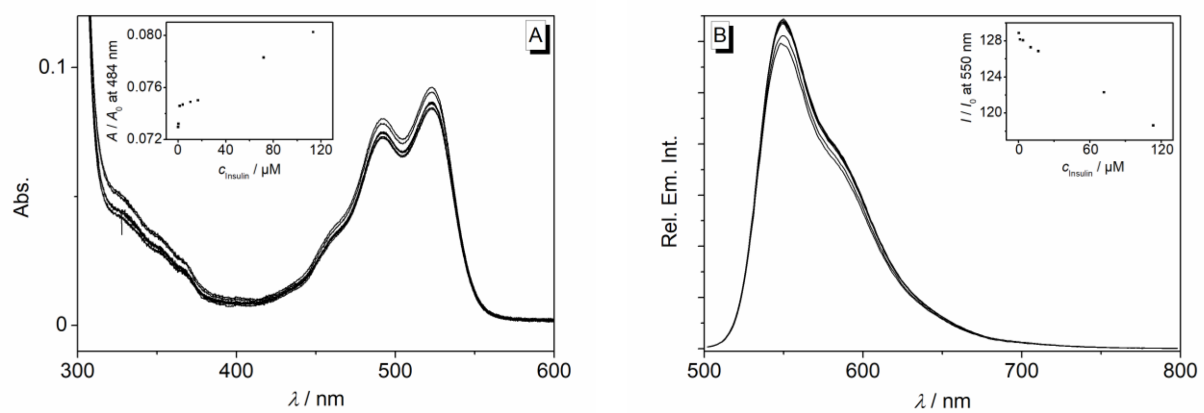

Figure S1. Photometric (A) and fluorimetric (B) titration of a **1**-**a2** mixture (c_{Ligand} = 20 µM, $c_{\mathbf{a2}}$ = 20 µM) with insulin in K-phosphate buffer (c_{K^+} = 25 mM, pH 7.0). Inset: Plot of absorption at 484 nm and emission at 550 nm *versus* DNA concentration.

The binding constant, K_b, was determined from the binding isotherm resulting from the fluorimetric titration (Figure S2) and fitting of the experimental data to the theoretical model according to equation 1 [2].

$$y = 0.5\,R \times \left(A + B + x - \sqrt{(A + B + x)^2 - 4Bx}\right) \qquad \text{(eq. 1)}$$

y = normalized emission intensity

A = B / K_b x c_{Ligand}

B = n / c_{Ligand}

x = c_{DNA}

Standard derivations (SD) of K_b values were calculated according to equation 2.

$$\text{SD}(K_b) = \{(\text{SD of } A \times K_b) / A\} \qquad \text{(eq. 2)}$$

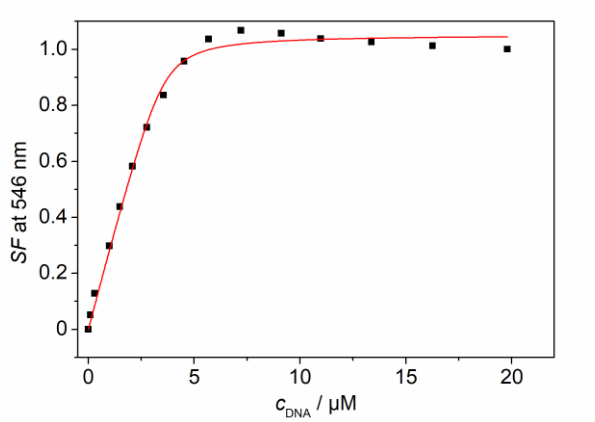

Figure S2. Fitting curve of the binding isotherm from the fluorimetric titration of **1** with **a2** for the determination of the binding constant (K_b). The red line represents the best fit of the experimental data to the theoretical model.

2. CD-spectroscopic analysis

CD spectra were recorded in K-phosphate buffer solution (c_{DNA} = 20.0 µM) at different ligand-DNA ratios (*LDR*). The CD measurements were performed at *LDR* = 0, 0.1, 0.2, 0.5, 1.0, 1.5, 2.0. CD signals were recorded with band width of 1 nm, recording speed of 1 nm/s and time per data point of 0.5 seconds. All samples were measured after an equilibration time of 1 h.

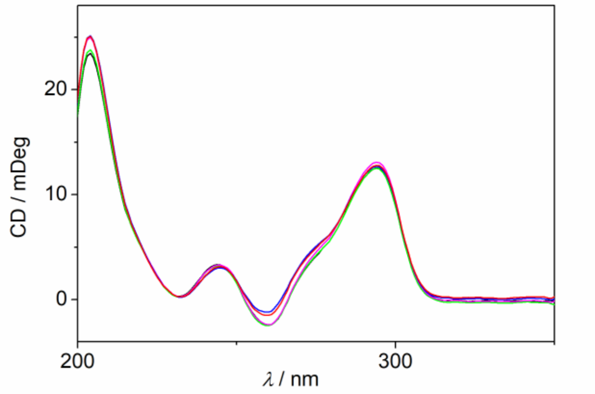

Figure S3. CD spectra of **a2** (c_{DNA} = 20 µM) in K-phosphate buffer (pH 7.0) at varying K^+ concentrations [c_{K^+} = 25 mM (black), 35 mM (green), 40 mM (magenta), 50 mM (blue), 100 mM (red)].

3. NMR-spectroscopic analysis

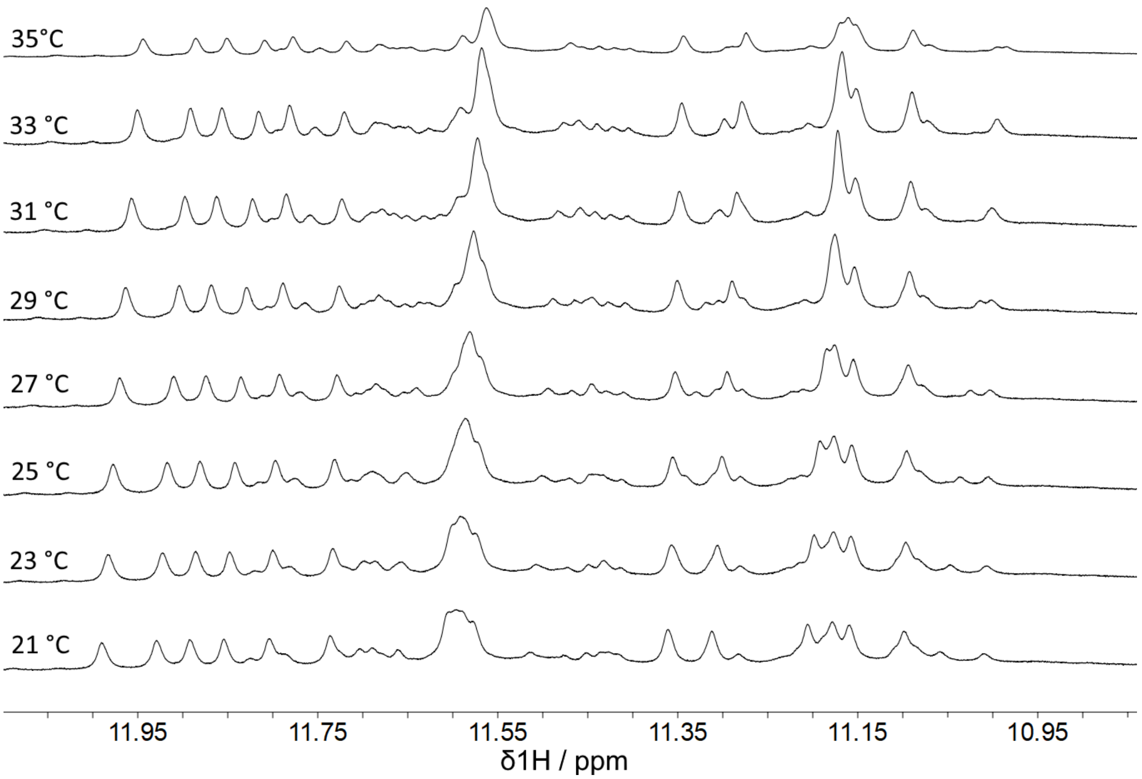

Figure S4. ^{1}H NMR spectra of **a2** (2.0 mM) in the range of the resonances of the guanine H1 protons at 10.9–12.0 ppm at different temperatures in K-phosphate buffer [c_{K^+} = 25 mM, pH 7.0, H_2O/D_2O (9:1), T = 25 °C].

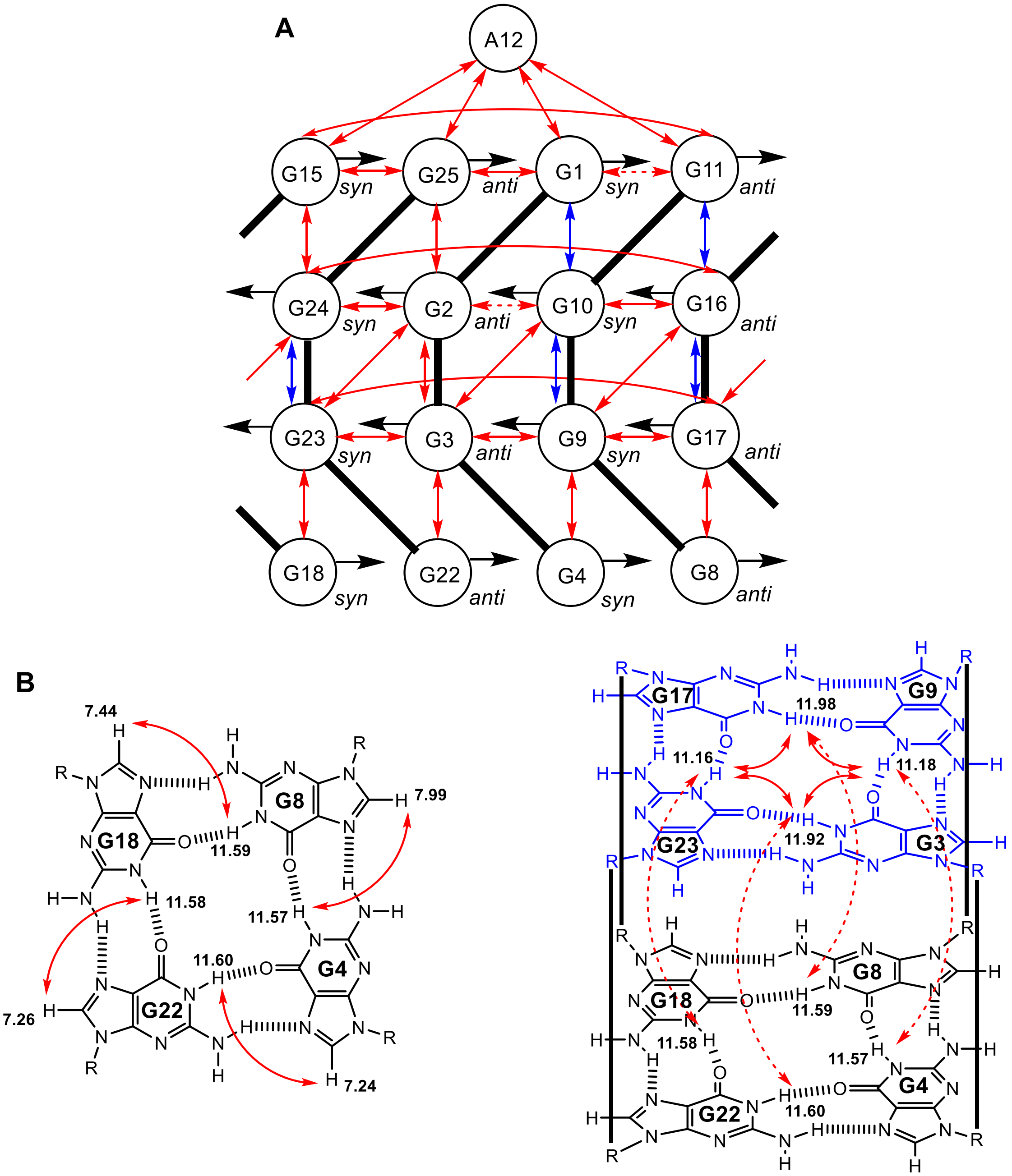

Figure S5. A: Schematic representation of the NOE signals between the guanine bases of **a2**; black: deoxyribose-phosphate backbone, red arrows: NOE signals; black arrows indicate the orientation of guanine H1 protons. The expected signals between G1/G10, G9/G10, G11/G16, G16/G17 and G23/G24 overlapped in the NOE spectrum and are depicted as blue arrows. B: Schematic representation of the observed interbase guanine H1/H8 signals as well as NOE signals between guanine H1 protons of the G4-G8-G18-G22 and G3-G9-G17-G23 quartets.

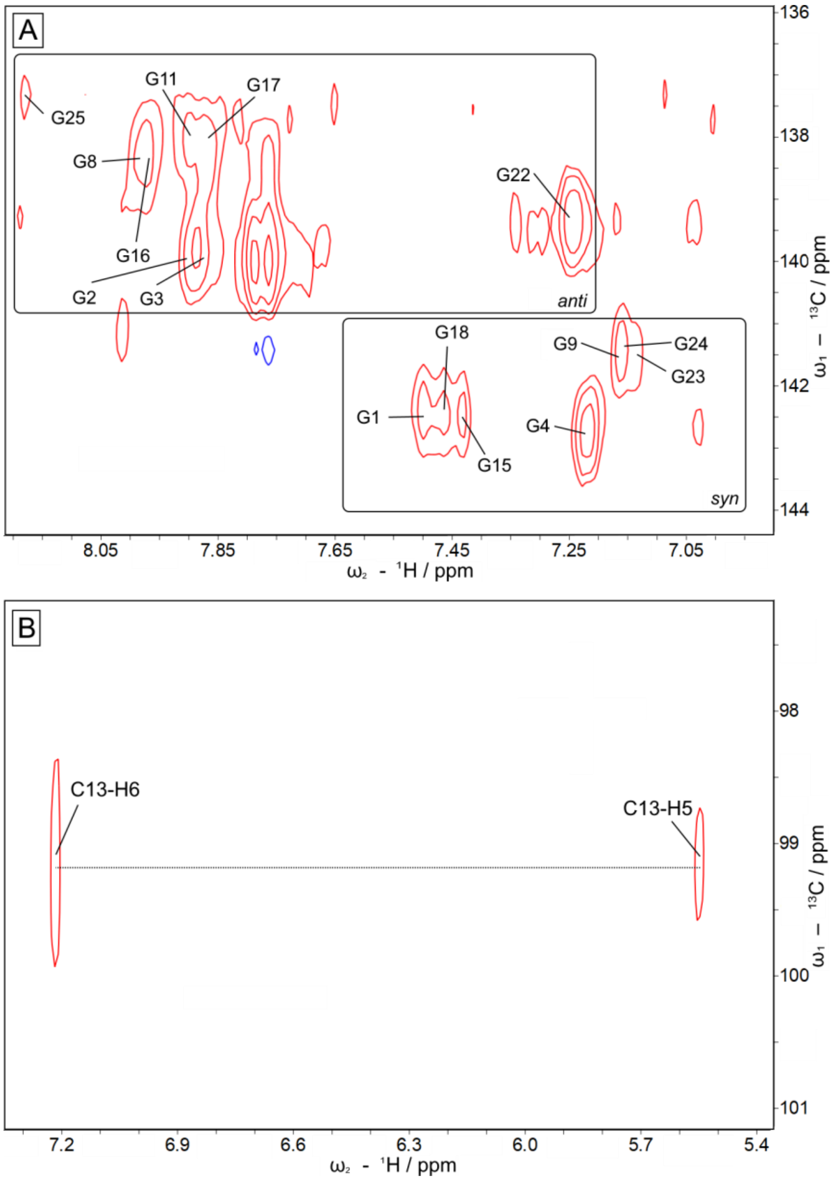

Figure S6. A: ^{1}H-^{13}C HSQC NMR spectrum of the resonances of guanine H8 and guanine C8 of **a2** (c_{DNA} = 2.0 mM) in K-phosphate buffer [c_{K^+} = 25 mM, pH 7.0, H_2O/D_2O (9:1), T = 25 °C]. B: Overlay of ^{1}H-^{13}C HSQC and ^{1}H-^{13}C HMBC NMR spectrum of the resonances of cytosine H5, H6 and C5 of **a2** (c_{DNA} = 2.0 mM) in K-phosphate buffer [c_{K^+} = 25 mM, pH 7.0, H_2O/D_2O (9:1), T = 25 °C].

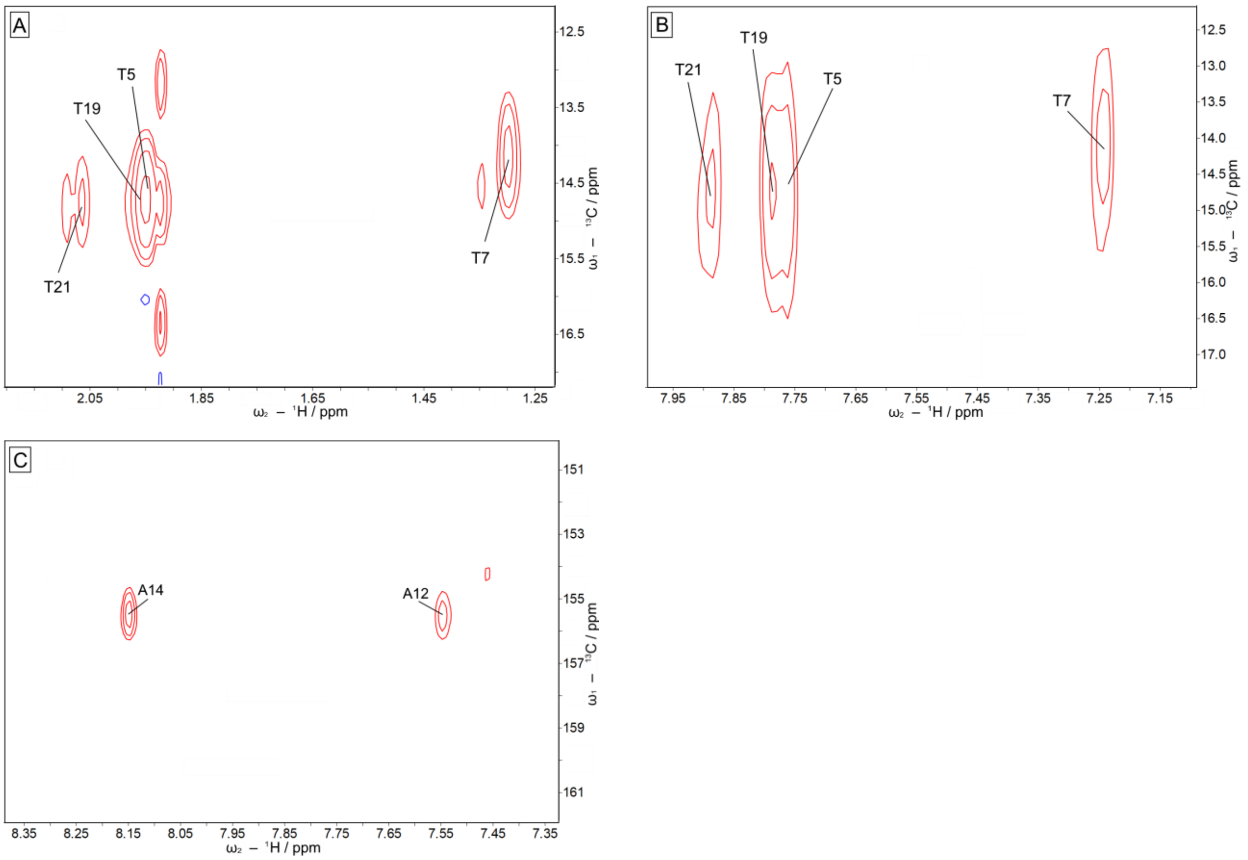

Figure S7. A: ^{1}H-^{13}C HSQC NMR spectrum of the resonances of thymine methyl groups (^{1}H and ^{13}C) (A) and of adenine H2 and C2 (C) of **a2** (c_{DNA} = 2.0 mM) in K-phosphate buffer [c_{K^+} = 25 mM, pH 7.0, H_2O/D_2O (9:1), T = 25 °C]. B: ^{1}H-^{13}C HMBC NMR spectrum of the resonances of thymine methyl groups (^{1}H and ^{13}C) of **a2** (c_{DNA} = 2.0 mM) in K-phosphate buffer [c_{K^+} = 25 mM, pH 7.0, H_2O/D_2O (9:1), T = 25 °C].

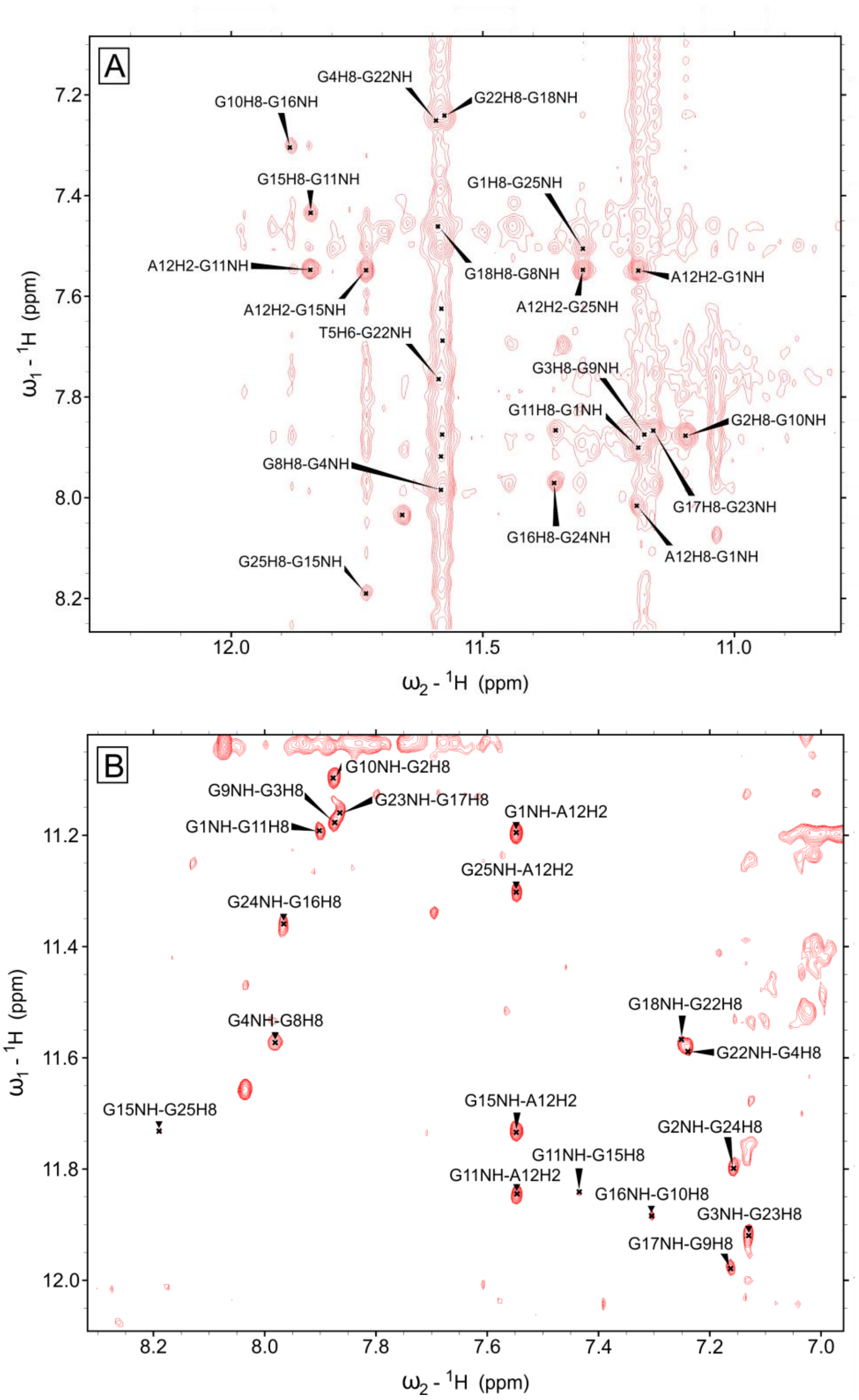

Figure S8. 2D NMR spectra (NOESY; A: range 7.1–8.2 vs 10.8–12.3; B: range 11.0–12.1 vs 6.9–8.3) of guanine imino and H8 protons of **a2** (c_{DNA} = 2.0 mM) in K-phosphate buffer [c_{K^+} = 25 mM, pH 7.0, H_2O/D_2O (9:1), T = 25 °C].

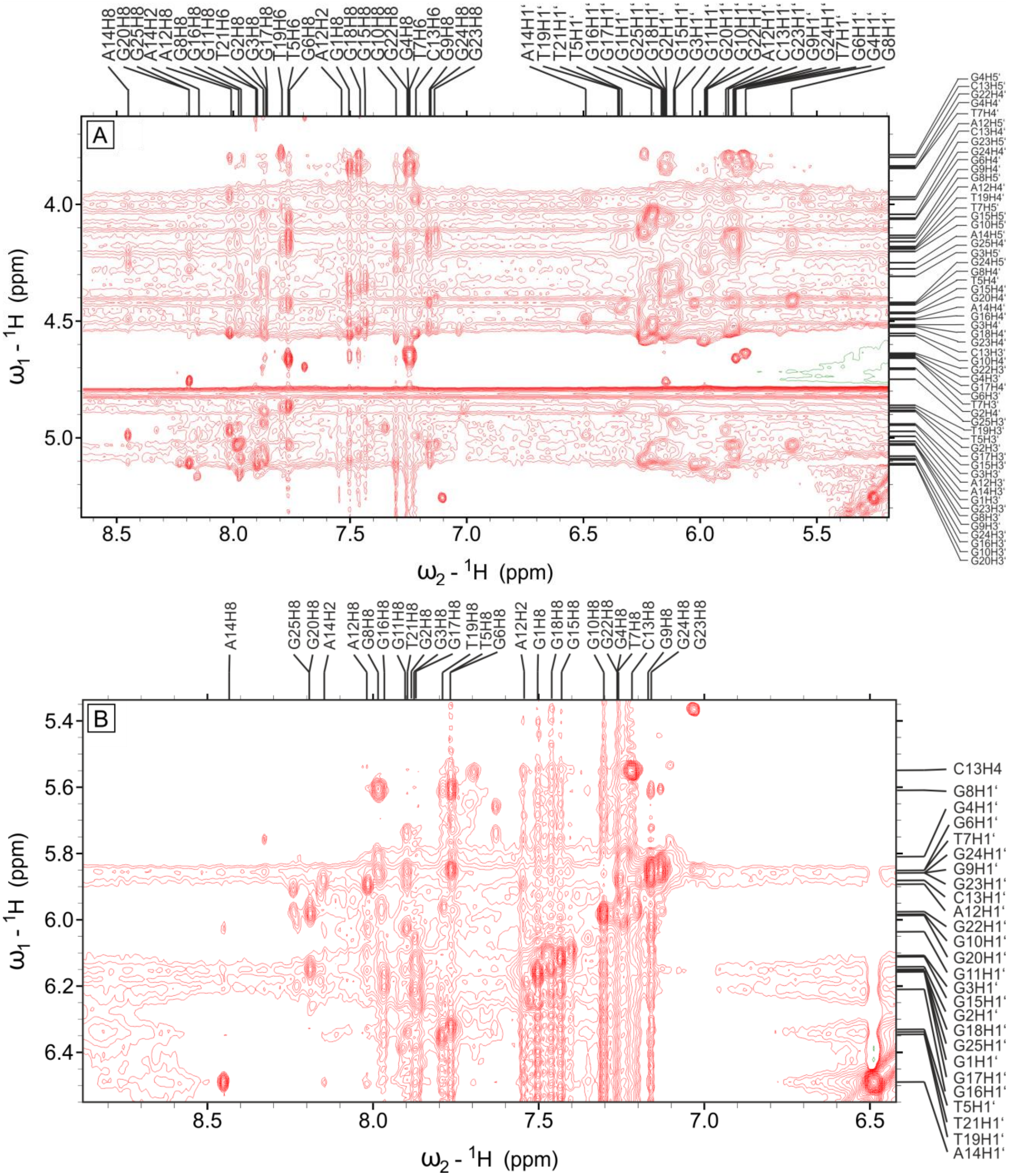

Figure S9. 2D NMR spectra (NOESY; A: range 4.6–5.3 vs 5.2–8.6; B: range 5.3–6.5 vs 6.4–8.9) of the aromatic and deoxyribose protons of **a2** (c_{DNA} = 2.0 mM) in K-phosphate buffer [c_{K^+} = 25 mM, pH 7.0, H_2O/D_2O (9:1), T = 25 °C].

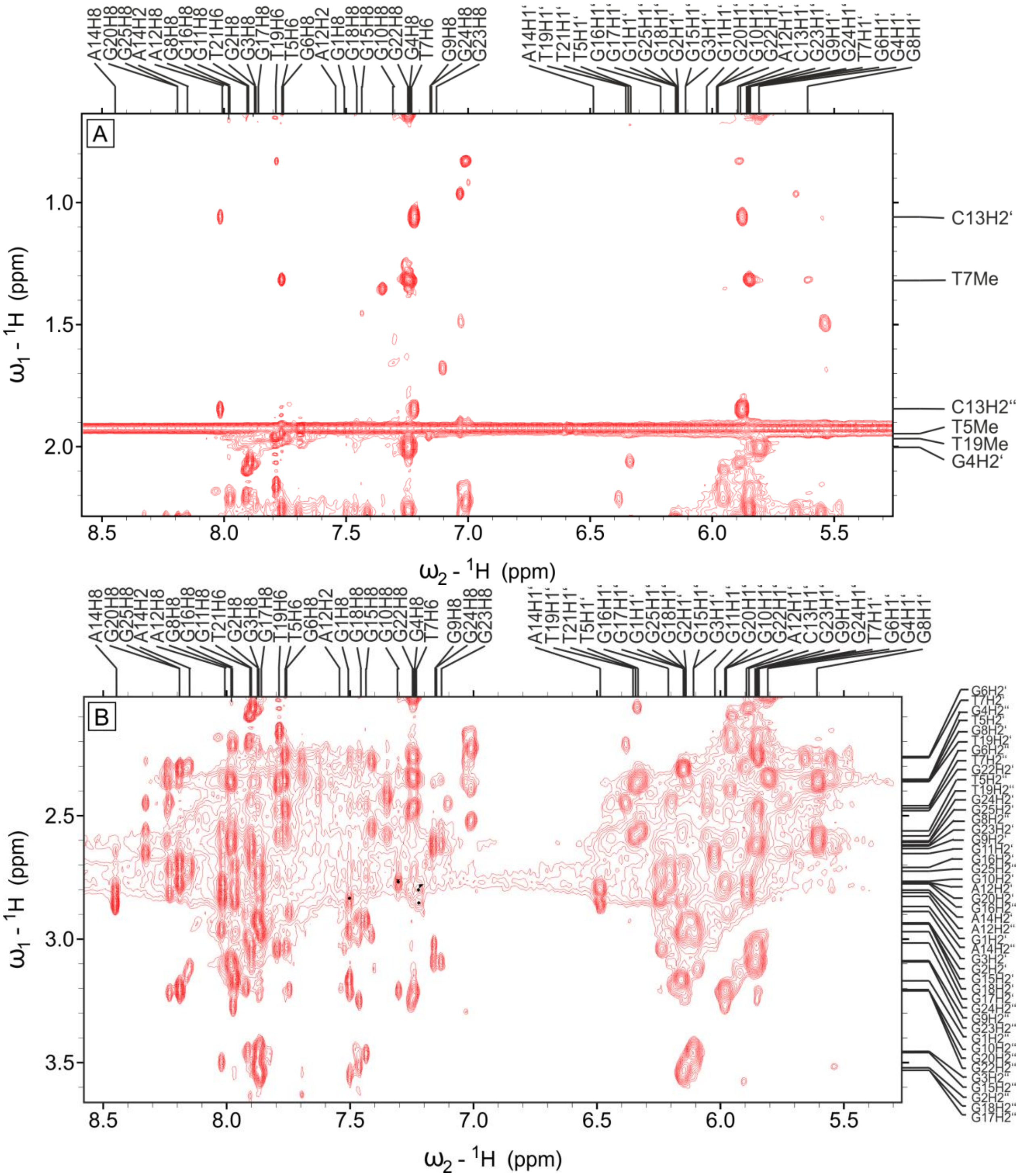

Figure S10. 2D NMR spectra (NOESY; A: range 0.7–2.3 vs 5.3–8.6; B: range 2.0–3.6 vs 5.3–8.6) of the aromatic and deoxyribose protons of **a2** (c_{DNA} = 2.0 mM) in K-phosphate buffer [c_{K^+} = 25 mM, pH 7.0, H_2O/D_2O (9:1), T = 25 °C].

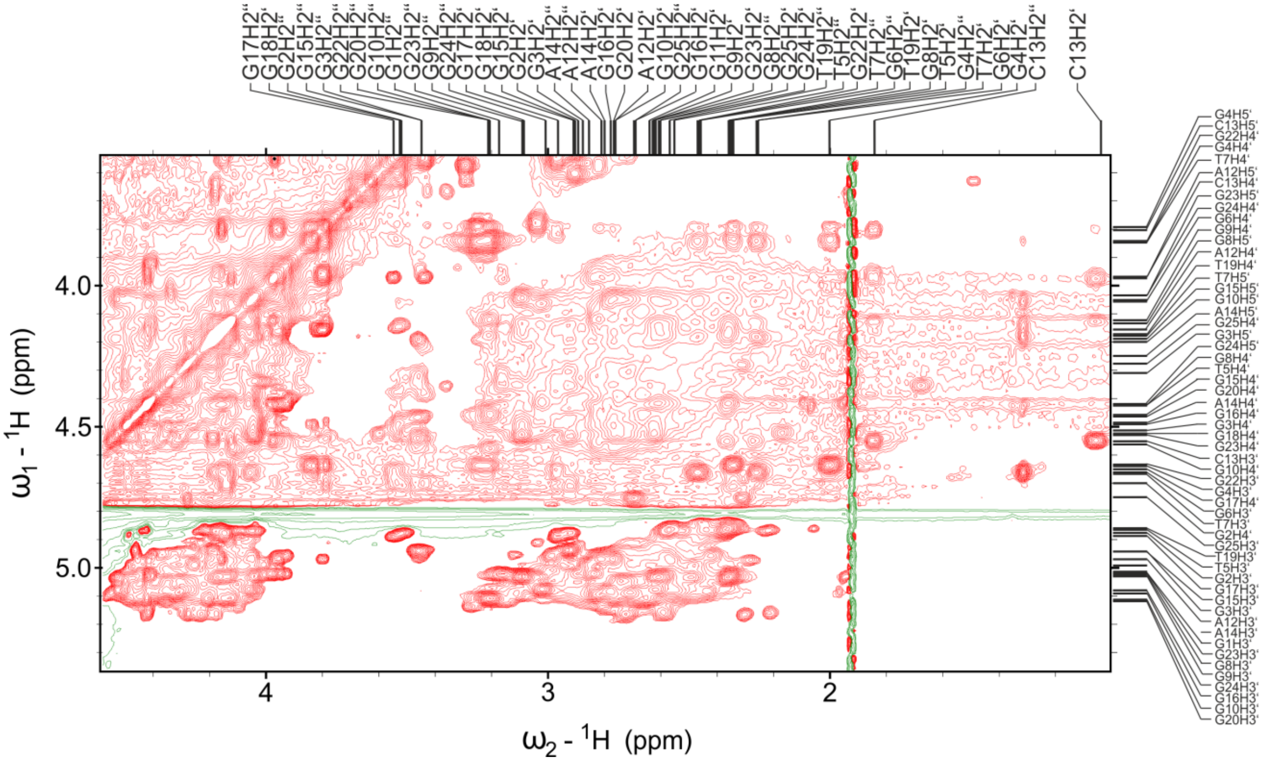

Figure S11. 2D NMR spectrum (NOESY) of the deoxyribose protons of **a2** (c_{DNA} = 2.0 mM) in K-phosphate buffer [c_{K^+} = 25 mM, pH 7.0, H_2O/D_2O (9:1), T = 25 °C].

Table S1. ^{1}H NMR chemical shifts of **a2**.[a]

Base	H1	H2	H5	H6	H8	Me	H1‘	H2‘/H2“	H3‘	H4‘	H5‘/H5“
$G1_{syn}$	11.19				7.50		6.16	2.84 / 3.17	5.02		
$G2_{anti}$	11.80				7.88		6.14	2.93 / 3.52	4.88	4.70[b]	
$G3_{anti}$	11.92				7.87		6.11	2.97/ 3.46	4.94	4.52[b]	4.31
$G4_{syn}$	11.57				7.24		5.81	2.00 / 2.35	4.64	3.84	3.79
T5				7.77		1.94	6.33	2.36 / 2.56	4.87	4.42[b]	
G6					7.76		5.85	2.26 / 2.46	4.66	4.06	
T7				7.24		1.32	5.85	2.26 / 2.47	4.66	3.84	4.18
$G8_{anti}$	11.59				7.99		5.61	2.36 / 2.61	5.03	4.42	4.14
$G9_{syn}$	11.18				7.16		5.86	2.63[b] / 3.09	5.03	4.13	
$G10_{syn}$	11.10				7.31		5.98	2.77 / 3.21	5.11	4.56	4.20[b]
$G11_{anti}$	11.84				7.90		6.03	2.65			
A12		7.55			8.02		5.89	2.77 / 2.81	4.97	4.16	3.97
C13			5.55	7.22			5.88	1.06 / 1.85	4.55	3.98	3.80
A14		8.15			8.45		6.49	2.80 / 2.86	4.99	4.49	4.25
$G15_{syn}$	11.73				7.44		6.11	2.93 / 3.46	4.94	4.46	4.19
$G16_{anti}$	11.88				7.97		6.21	2.71 / 2.78	5.09	4.49[b]	
$G17_{anti}$	11.98				7.86		6.16	2.97 / 3.55	4.89	4.65	
$G18_{syn}$	11.58				7.46		6.15	2.93 / 3.52	5.07[b]	4.53	
T19				7.79		1.96	6.35	2.37 / 2.58	4.87	4.18	
G20					8.19		5.98	2.77 / 3.21	5.11	4.46	
T21				7.89		2.06	6.34[b]				
$G22_{anti}$	11.60				7.25		5.85[b]	2.48 / 3.25	4.64	3.84	
$G23_{syn}$	11.16				7.14		5.86	2.62 / 3.09	5.03	4.53	4.04
$G24_{syn}$	11.36				7.16		5.85	2.60[b] / 3.02	5.08	4.06	4.42
$G25_{anti}$	11.30				8.19		6.15	2.31 / 2.70	4.75	4.28	

[a] Dashed horizontal lines separate the different quartet units. [b] No unambiguous assignment due to overlapping signals

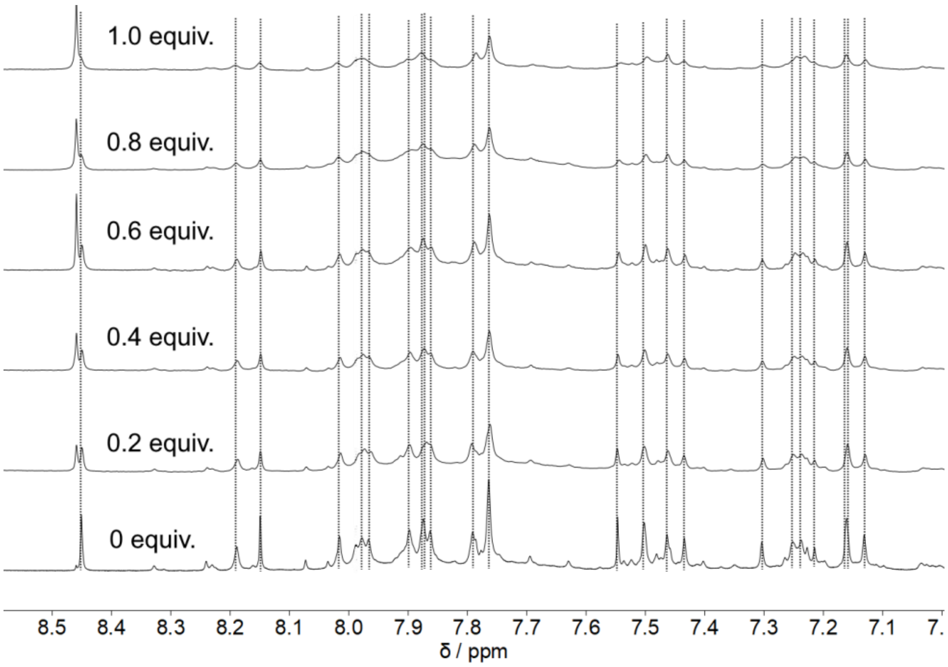

Figure S12. ^{1}H NMR spectrum of **a2** (c_{DNA} = 2.0 mM) in the range of the aromatic protons (7.0–8.5 ppm) with increasing amount of ligand **1** in K-phosphate buffer (c_{K^+} = 25 mM, pH 7.0, T = 25 °C) before (lower panel) and directly after exchange of the solvent from H_2O/D_2O (9/1) to D_2O (upper panel).

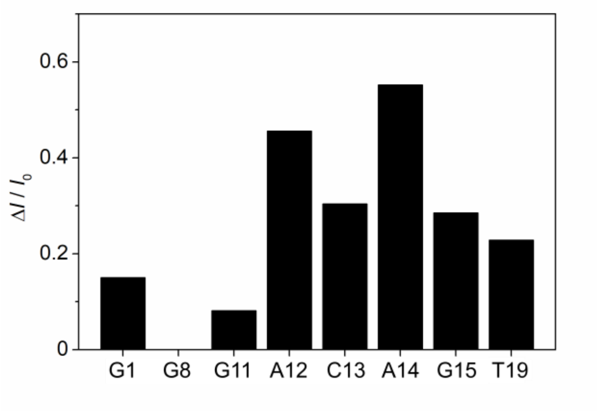

Figure S13. Relative changes of the ^{1}H NMR signal intensities of the aromatic protons upon addition of 0.6 molar equivalents of ligand **1**; signal intensities were determined relative to the one of G8.

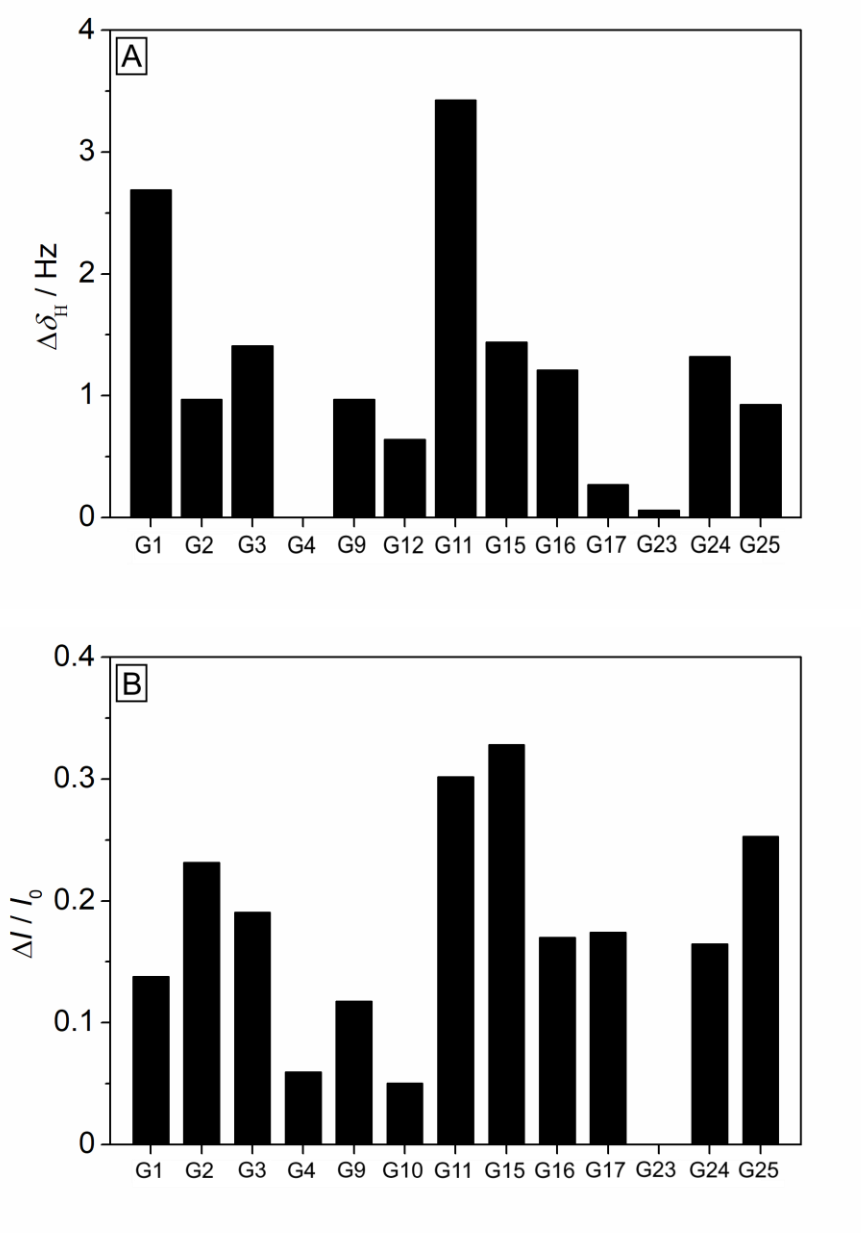

Figure S14. Shift (A) and changes of the relative signal intensities (B) of the ^{1}H NMR signal of the guanine H1 protons upon addition of 0.6 molar equivalents of ligand **1**. Signal intensities were determined relative to the one of G23. The signal intensities and chemical shift changes of G4 were determined from the maximum of the overlapped signals of G4, G8, G18 and G22.

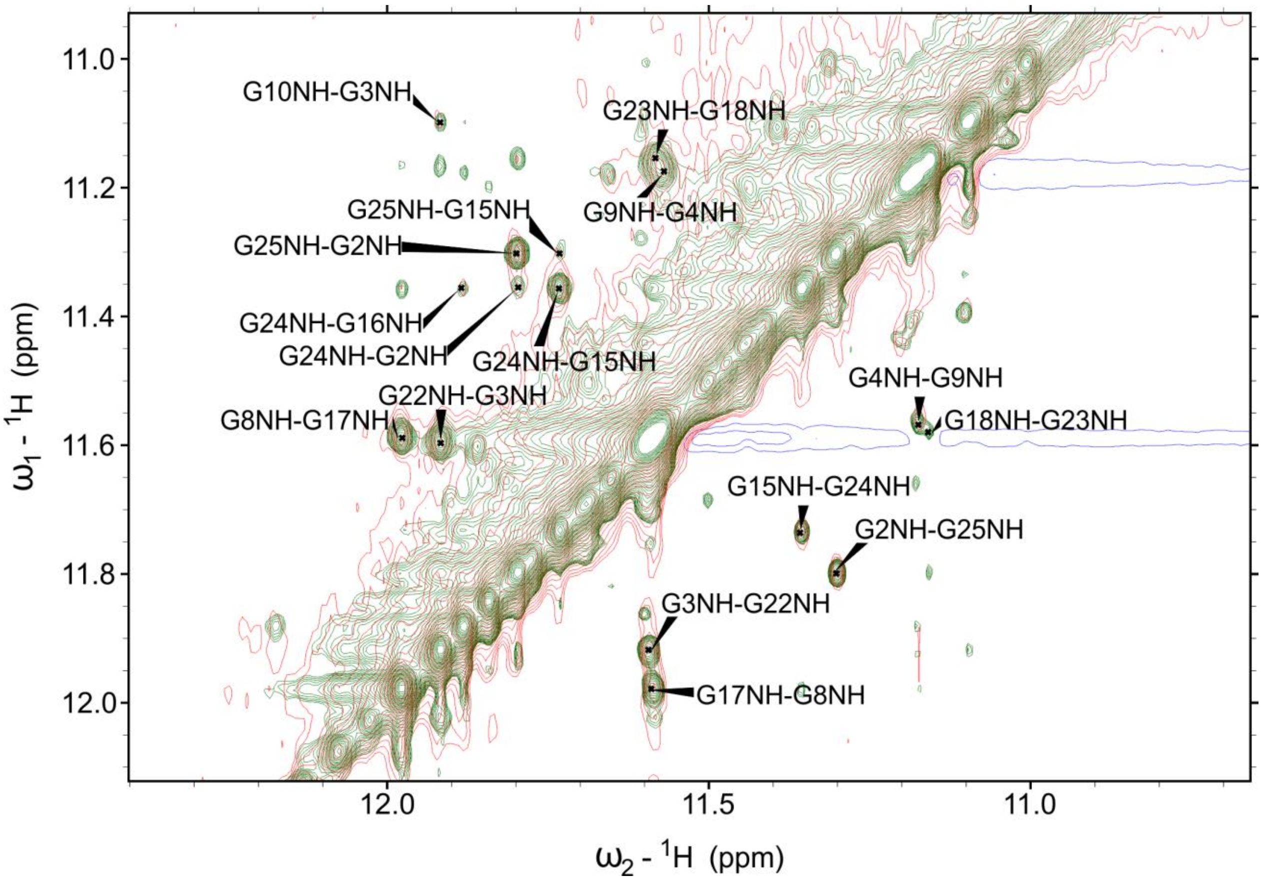

Figure S15. Superposed 2D NMR spectra (NOESY) of guanine imino protons of **a2** (c_{DNA} = 2.0 mM) in the absence (green) and presence (red) of 0.6 molar equivalents of ligand **1** in K-phosphate buffer [c_{K^+} = 25 mM, pH 7.0, H_2O/D_2O (9:1), T = 25 °C].

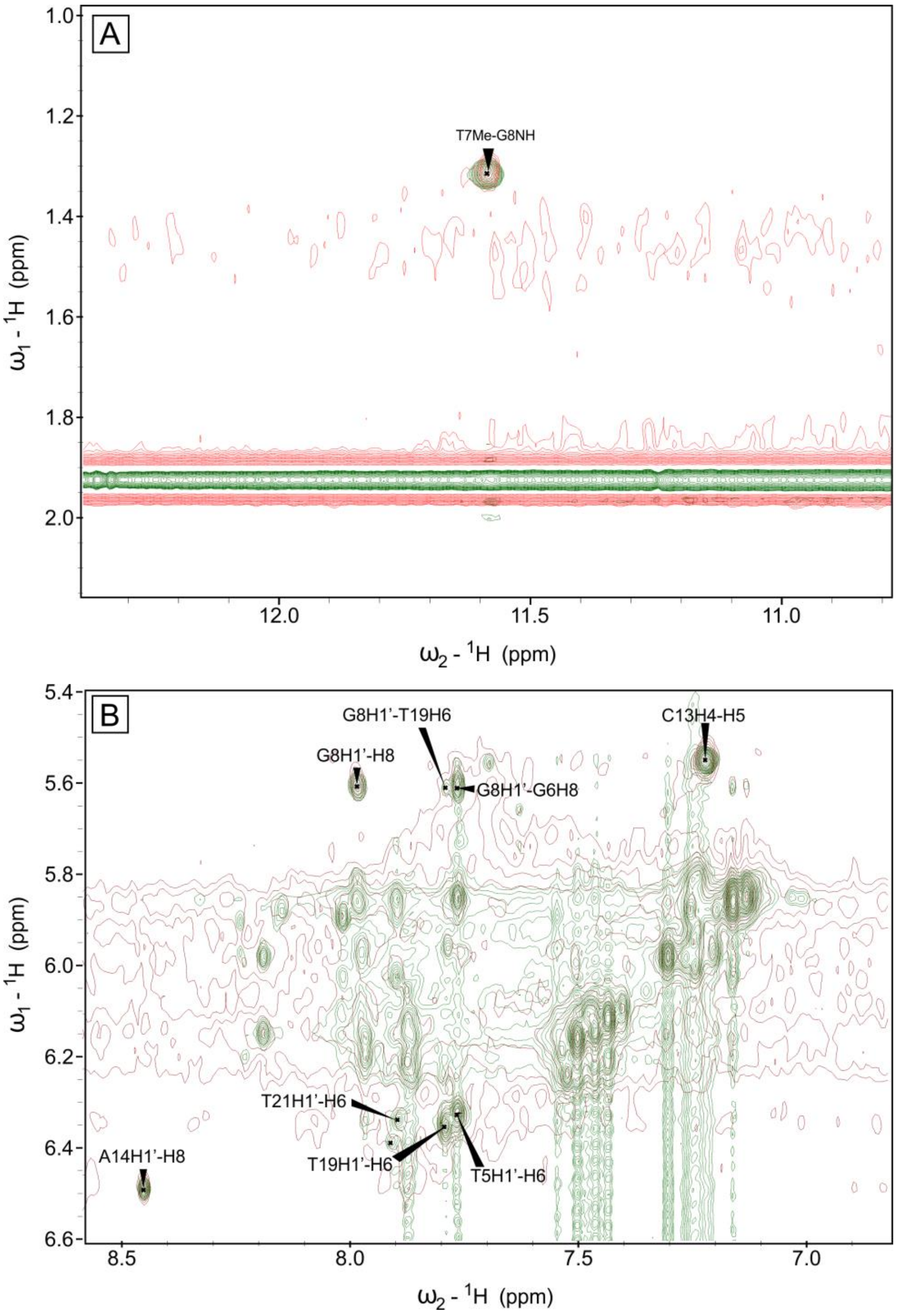

Figure S16. Superposed 2D NMR spectra (NOESY) of the region of guanine imino and thymine methyl protons (A) and aromatic and deoxyribose protons (B) of **a2** (c_{DNA} = 2.0 mM) in the absence (green) and presence (red) of 0.6 molar equivalents of ligand **1** in K-phosphate buffer [c_{K^+} = 25 mM, pH 7.0, H_2O/D_2O (9:1), T = 25 °C].

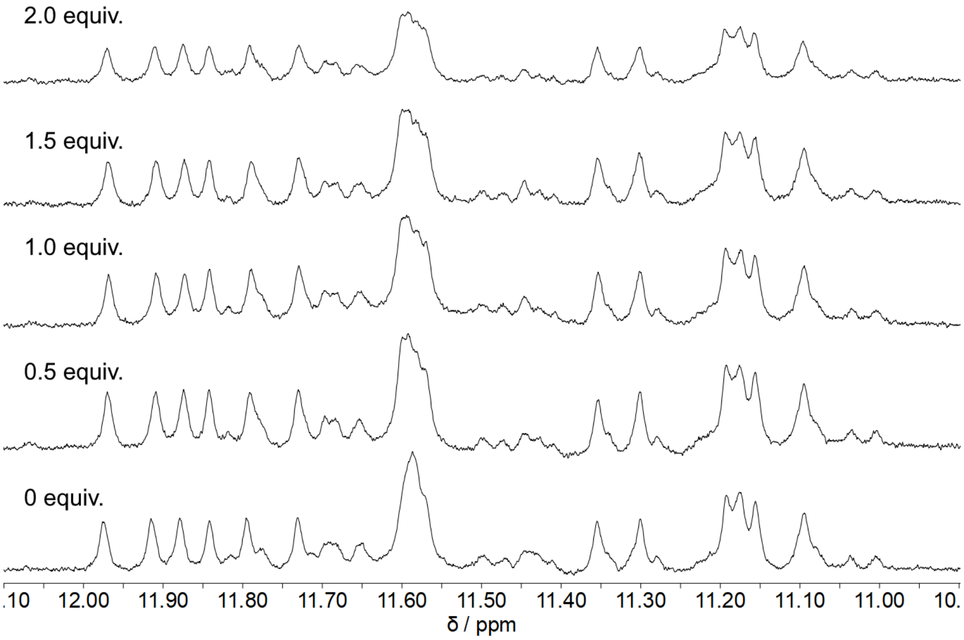

Figure S17. ^{1}H NMR spectrum of **a2** (c_{DNA} = 2.0 mM) in the range of the guanine imino protons (11.0–12.0 ppm) with increasing amount of insulin in K-phosphate buffer (c_{K^+} = 25 mM, pH 7.0, T = 25 °C).

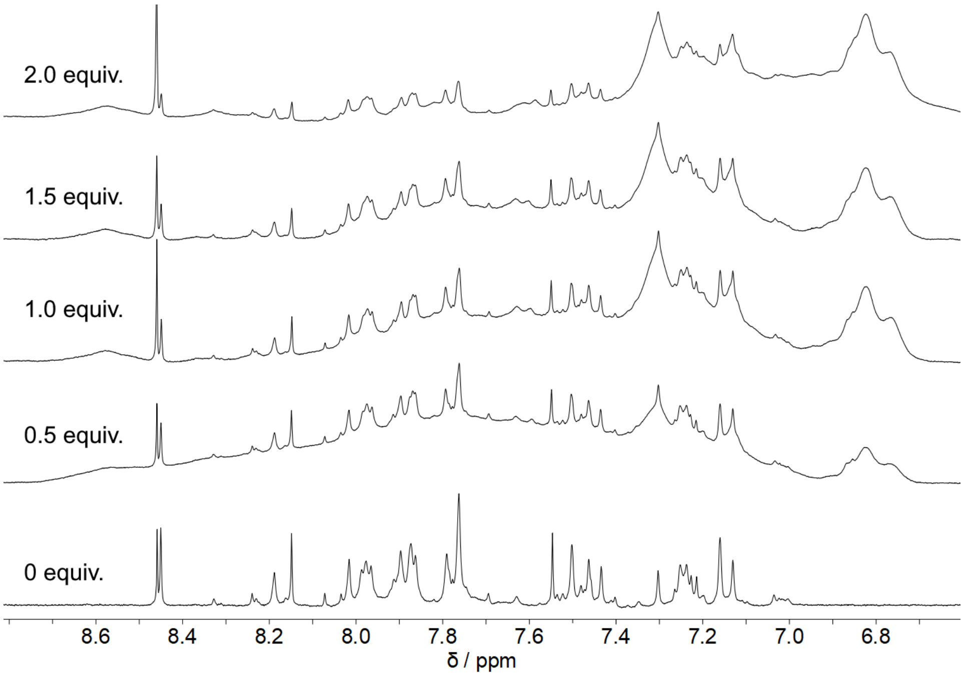

Figure S18. ^{1}H NMR spectrum of **a2** (c_{DNA} = 2.0 mM) in the range of the aromatic protons (6.70–8.70 ppm) with increasing amount of insulin in K-phosphate buffer (c_{K^+} = 25 mM, pH 7.0, T = 25 °C).

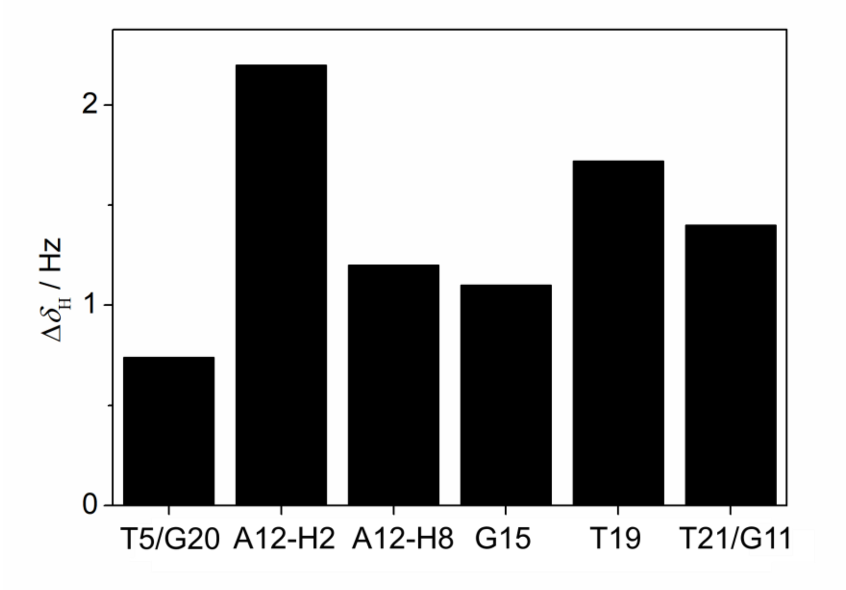

Figure S19. Shift of the ^{1}H NMR signal intensities of the guanine H1 protons upon addition of 1.5 molar equivalents of insulin. The signals of T5 and G20 as well as T21 and G11 overlapped.

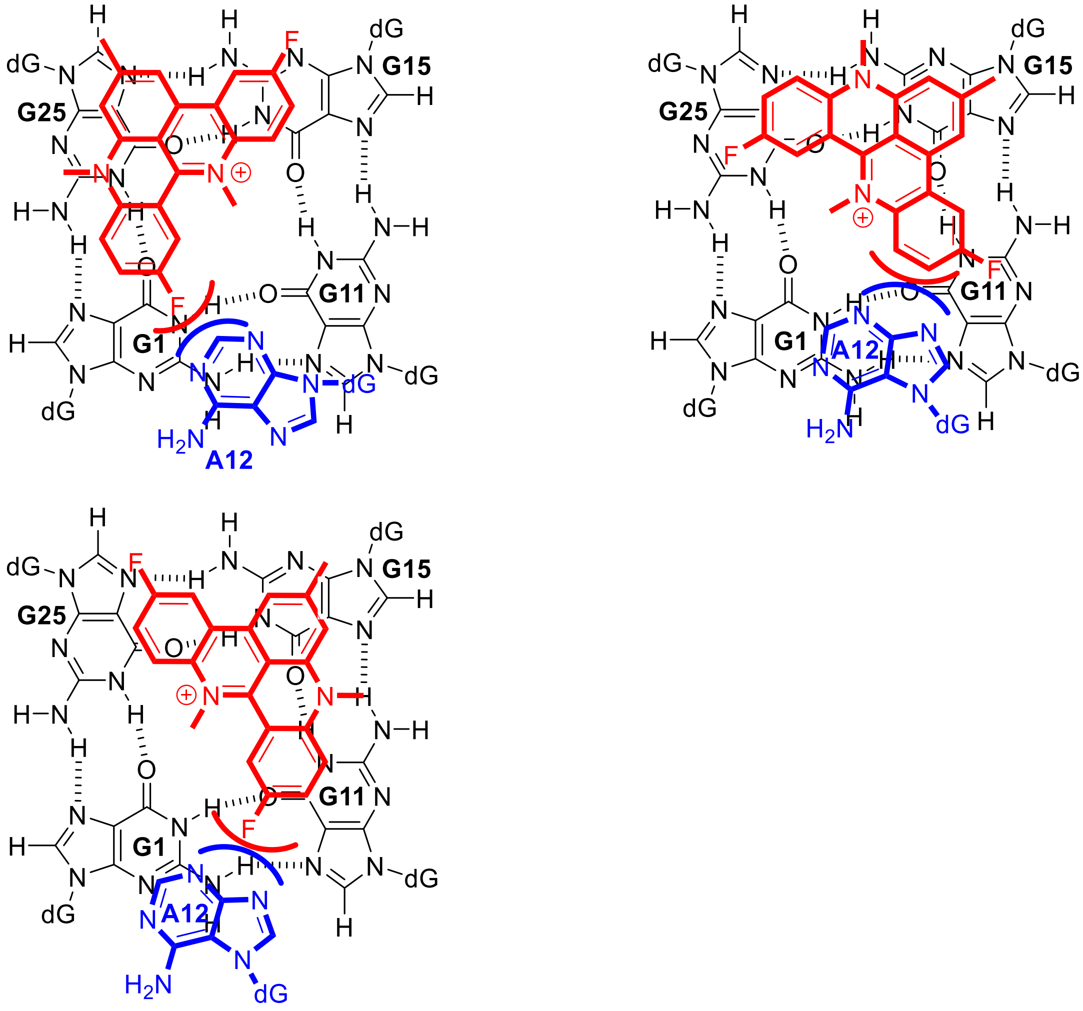

Figure S20. Binding modes of **1** to **a2** which were proposed to be less favorable due to steric interactions between ligand **1** and A12.

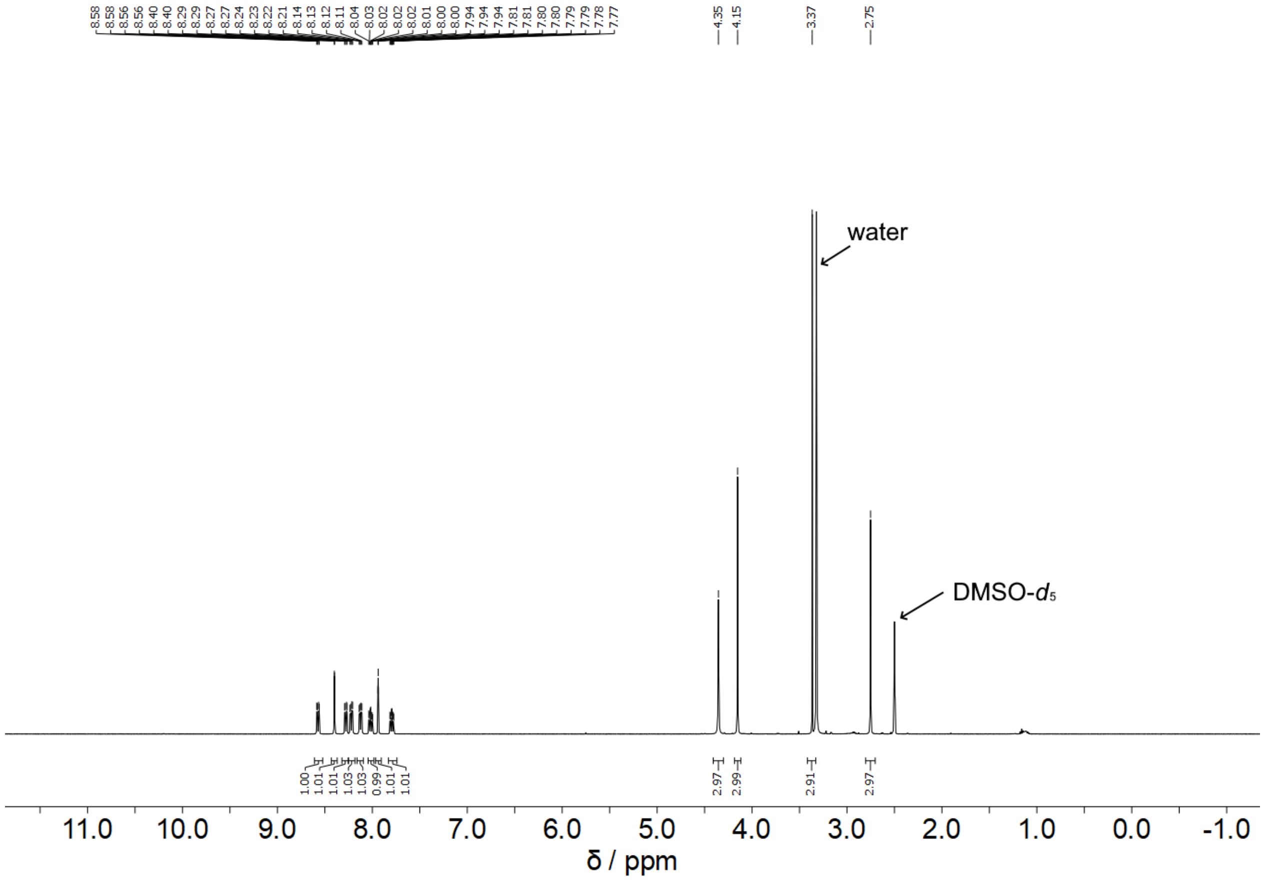

Figure S21. ^{1}H NMR spectrum (500 MHz) of **1** in DMSO-d_6.

4. References

1. Bortolozzi, R.; Ihmels, H.; Thomas, L.; Tian, M.; Viola, G. 9-(4-Dimethylaminophenyl)-benzo[*b*]quinolizinium: A Near-Infrared Fluorophore for the Multicolor Analysis of Proteins and Nucleic Acids in Living Cells. *Chem. Eur. J.* **2013,** *19,* pp. 8736–8741.

2. Stootman, F. H.; Fisher, D. M.; Rodger, A.; Aldrich-Wright, J. R. Improved curve fitting procedures to determine equilibrium binding constants. *Analyst* **2006**, *131*, pp. 1145–1151.
